# Angiotensin II-induced cardiac fibrosis and dysfunction are exacerbated by deletion of cGKI in *periostin* ^+^ myofibroblasts

**DOI:** 10.1042/CS20241204

**Published:** 2025-05-28

**Authors:** Melanie Cruz Santos, Lena Birkenfeld, Thomas Pham, Selina Maier, Katharina Paulus, Lena Ullemeyer, Amelie Knauer, Clement Kabagema-Bilan, Natalie Längst, Anna Roslan, Nina Wettschureck, Meinrad Gawaz, Fumito Ichinose, Robert Lukowski

**Affiliations:** 1 Department of Pharmacology, Toxicology and Clinical Pharmacy, Institute of Pharmacy, University of Tübingen, Tübingen, Germany; 2 Department of Pharmacology, Max-Planck-Institute for Heart and Lung Research, Bad Nauheim, Germany; 3 Department of Cardiology and Angiology, University of Tübingen, University Hospital Tübingen, Tübingen, Germany; 4 Harvard Medical School, Boston, MA, U.S.A.; 5 Department of Anesthesia, Critical Care and Pain Medicine, Massachusetts General Hospital, Boston, MA, U.S.A.

**Keywords:** angiotensin II, cardiac myofibroblasts, cardiac remodeling, cGKI, cGMP, fibrosis, periostin, PKG

## Abstract

Differentiation of cardiac fibroblasts (CFs) into myofibroblasts (CMFs) is considered a critical event in response to the maladaptive cardiac remodeling triggered by angiotensin II (Ang II). Active CMFs are proliferative and contribute to the production of extracellular matrix and matricellular proteins such as periostin, to myocardial fibrosis, and thus to muscle stiffness. Although previous studies provided substantial evidence for the antifibrotic signaling elicited by NO/NP-cGMP-cGKI, the role of this axis in modulating CMF function(s) *in vivo* remains unclear. To address this, Ang II was delivered through osmotic minipumps into tamoxifen-induced CMF-specific cGKI knockout (*cmf*KO) and littermate control (CTR) male mice. CMF-restricted Cre activity in *periostin* ^+^ cells resulted in an effective depletion of the cGKI protein observed in myocardial sections and in primary CF/CMF protein lysates obtained from Ang II- and tamoxifen-treated *cmf*KO. Although both genotypes responded identically to Ang II in terms of blood pressure (BP) and cardiac enlargement, *cmf*KO hearts showed significantly increased cardiomyocyte cross-sectional areas and developed a marked increase in myocardial fibrosis. Moreover, non-invasive echocardiography revealed a structure-related distortion of global systolic function and longitudinal deformation capacity in *cmf*KO *versus* CTR. Consistent with the results obtained *in vivo*, we observed a higher proliferation rate of CF/CMF derived from Ang II-treated *cmf*KO hearts compared with respective CTR cells, as well as an increase in cardiomyocyte apoptosis in the absence of cGKI in *periostin* ^+^ CMF. Our data confirm that endogenous cGKI function in *periostin* ^+^ CMFs counteracts the Ang II-induced morphologic and structural changes that impair cardiomyocyte survival ultimately causing loss of heart function in mice.

## Introduction

Myocardial fibrosis, defined by excessive deposition of extracellular matrix (ECM) proteins, leads to fibrotic scarring of the myocardium and is a common pathologic feature associated with a wide spectrum of cardiovascular diseases (CVDs), including hypertensive heart disease, diabetic hypertrophic, and idiopathic dilated cardiomyopathy [1-4], which, together with other CVDs, are the leading cause of death worldwide [5,6]. Although scar formation, especially after myocardial infarction (MI), compensates for cardiomyocyte (CM) cell death and prevents myocardial rupture, the progression of myocardial fibrosis is associated with a decline in cardiac function, frequent arrhythmias, loss of cardiac elasticity, and eventually resulting in the development of heart failure (HF) with poor outcomes [7-10].

Extensive preclinical studies have identified cardiac myofibroblasts (CMFs) as the major effector cell type contributing to the structural and functional changes of the heart in response to stress [11,12]. CMFs do not appear in the healthy heart but instead arise from activated resident cardiac fibroblasts (CFs) following cardiac injury. Accordingly, MI or a chronic elevation of the angiotensin II (Ang II) levels [11,13-15], among other causes, frequently triggers myocardial fibrosis and also CM hypertrophy, which is another feature of the structural, geometrical, and functional remodeling process.

Ang II is one major effector peptide of the renin–angiotensin–aldosterone system (RAAS) that conveys its adverse actions in the cardiovascular system mainly via the AT_1_-receptor subtype, which is found at high density in CMs [16] and blood vessels [17,18], as well as in cultured human and rodent CFs [19-21]. Ang II effectively stimulates the proliferation and migration of CFs, highlighting its potential to activate CFs to phenotypically and functionally ‘switch’ to CMFs [14,19,20,22,23]. Interestingly, Ang II infused chronically at pressor dosages induces a change in the phenotype of CFs, resulting in the accumulation of ECM in the myocardium within days of treatment [24]. However, additional cell types including pericytes, mesenchymal, and hematopoietic cells have been suggested as possible sources for CMFs in the injured heart, leading to an ongoing debate about the actual progenitor cell type of CMFs [8,13]. Interestingly, CMFs combine the cytoskeletal and contractile functions of vascular smooth muscle cells (VSMCs) with the ECM-producing abilities of CFs. At the molecular level, this results in high expression levels of the cytoskeletal protein alpha smooth muscle actin (α-SMA) and an excessive synthesis and secretion of ECM proteins such as type I and III collagen (COL I and COL III), fibronectin (FN1), and matricellular proteins like periostin (POSTN), which together contribute to the increase in chamber stiffness, impaired muscle relaxation, and ultimately in a decline in cardiac performance [25-28]. Given these features of CMFs, a modulation of their function should be a promising strategy to counteract or even prevent the development and progression of multiple heart diseases, especially HF.

The second messenger 3′,5′-cyclic guanosine monophosphate (cGMP) signaling axis is a key regulator of cardiovascular homeostasis [29-31]. Cyclic GMP is generated intracellularly either by nitric oxide (NO)-mediated activation of NO-sensitive (soluble) guanylyl cyclase (NO-GC *aka* GC or sGC) isoform 1 or 2 or by natriuretic peptides (NP)-dependent stimulation of membrane-bound particulate guanylyl cyclase (pGC) receptors, also designated GC-A and GC-B [32,33]. The resulting increase in cGMP in the heart causes activation of the cGMP-dependent protein kinase type I (cGKI), which is considered to be the major downstream effector of the NO/NP-cGMP cascade in multiple cardiac cell types [34,35]. Another key element of this cascade are cGMP-hydrolyzing phosphodiesterases (PDEs), which control the duration, amplitude, and spread of an intracellular cGMP signal. PDEs, that can hydrolyze cAMP in addition to cGMP, are responsible for the so-called cross-talk between this two signaling pathways [35].

Clinical implementation of sacubitril, a neprilysin inhibitor that prevents, besides other effects, the degradation of NPs, and vericiguat, a NO-GC stimulator, in the treatment of HF with reduced ejection fraction (HFrEF) emphasize that the cGMP elevation presumably resulting from these principles improves patient outcomes. However, neither the exact cell type(s) nor the downstream mechanisms targeted by both drugs are clear [36-38]. Besides the widely appreciated effects of cGMP on the survival and growth of CMs [39,40], growing evidence from cell- and animal-based studies supports the notion that cGMP counteracts the adverse cardiac remodeling process in HF models by modulating the activity and phenotype of CFs and/or CMFs [41-43]. Accordingly, the analysis of genetically modified mice globally lacking NO-GC1 [44], which is the major NO-GC isoform in the cardiovascular system, or cGKI specifically in CMs [45] showed a significant increase in collagen deposition due to chronic Ang II challenge. Moreover, a CM-specific deletion of the Ca^2+^- and voltage-activated K^+^ channel BK, an established downstream target of cGMP/cGKI signaling, caused an unusually high accumulation of fibrosis in post-MI hearts [46]. Sildenafil, a PDE5 inhibitor with widely reported anti-hypertrophic and anti-fibrotic potential [47], counteracted the Ang II-induced increase in collagen deposition and fibrotic marker gene expression but only when cGKI was present in all non-smooth muscle cell types of the heart [48]. Among other possible explanations, the latter study strongly suggests that CF/CMF cGKI signaling may be involved in mediating the beneficial effects of the PDE5/cGMP axis on fibrosis. In contrast, however, chronic cGKI activation, caused by a constitutive, cGMP-independent regulation of the kinase in mice, can be harmful to the heart, especially in the presence of Ang II [49].

Taken together, these studies imply a strong anti-fibrotic function for the endogenous cardiac cGMP/cGKI axis. However, due to the lack of suitable models, it is mechanistically still unclear how or whether the signaling pathway counteracts the detrimental remodeling processes through a function that originates directly from CFs and/or CMFs [50-54].

To close this gap in knowledge, the present study aimed to explore the role of cGKI in *Postn*-positive (*Postn* ^+^) CMFs, as *Postn* ^+^ CMFs have been identified as the key effector cells contributing to myocardial fibrosis in neurohumoral, i.e., Ang II-induced cardiac stress models [13]. To generate CMF-specific conditional cGKI knockout (*cmf*KO) and corresponding control (CTR) littermates, we intercrossed floxed cGKI mice to a tamoxifen (TAM)-inducible *Postn*iCre strain [50]. This strategy allowed us to investigate putative functions of cGKI in a defined CMF population that develops exclusively in response of the heart to pathological stresses, while prior to the TAM challenge, ‘pre-mutant’ cGKI animals should develop normally. By assessing cardiac outcome in terms of fibrosis development, extent of hypertrophy, and multiple determinants of cardiac function, as well as by monitoring the proliferative behavior of isolated CF/CMF primary cell cultures, we ultimately wanted to clarify if the cGMP/cGKI signaling cascade in CMFs affects the adverse remodeling induced by chronic Ang II exposure.

### Methods

The online supplement contains further descriptions of the materials and methods.

### Animals

All animal experiments performed were authorized by the local Ethics Committee for Animal Experiments (Regierungspräsidium Tübingen, PZ02/22 G) and are in compliance with the European Directive 2010/63/EU on the protection of animals used in scientific research. Animals were housed in a standardized cage system at defined room temperature (RT) and humidity conditions and had *ad libitum* access to food and water during a cycle of alternating 12 h of light and 12 h of darkness.

To inactivate cGKI specifically in the CMF cell population, a transgenic *Postn*iCre^tg/+^ (Tg(Postn-icre/ERT2)#Wet) mouse line was employed expressing a TAM-inducible Cre recombinase under the control of *Postn* promoter [50]. CMF-specific conditional cGKI knockout (*cmf*KO; genotype: *Postn*iCre^tg/+^ x cGKI^fl/fl^) and corresponding littermate controls (CTR; genotype: *Postn*iCre^tg/+^ x cGKI^+/+^) were obtained by intercrossing *Postn*iCre^tg/+^ to heterozygous floxed cGKI (cGKI^fl/+^) [55-58] parental animals. Male mice, weighing 20–30 g and aged 10–16 weeks, were used for experiments. Tissue specificity and efficiency of Cre-recombination were assessed by intercrossing the transgenic *Postn*iCre^tg/+^ mouse line with the ROSA^mT/mG^ (*B6.129(Cg)-Gt(ROSA)26Sor^tm4(ACTB-tdTomato,-EGFP)Luo^/J*) double-fluorescent Cre reporter mouse line [59], resulting in the generation of experimental 10- to 16-week-old male double-transgenic ROSA^mT/mG^ × *Postn*iCre^tg/+^ and corresponding ROSA^mT/mG^ control mice.

For post-mortem analyses, experimental mice were euthanized in their home cage with CO_2_ or under deep isoflurane anesthesia followed by cervical dislocation. All animal studies are reported in compliance with the ARRIVE guidelines [60].

### Sex as a biological variable

Male mice, weighing 20–30 g and aged 10–16 weeks, were used for experiments. Our study examined male mice to avoid potential interactions between the female sex hormones in our tamoxifen-inducible model and because male animals usually exhibited less variability in the cardiac stress response. The pathophysiological effects of Ang II on the structure and function of the left ventricle have been extensively studied in male mice. Also, by focusing on one sex, the total number of subjects included in the study was lower, but this clearly limits any conclusions of our study for the female gender.

### Implantation of osmotic minipumps

For induction of cardiac hypertrophy and cardiac fibrosis, osmotic minipumps (ALZET^®^ pump model 1004 #0009922) releasing Ang II (Sigma-Aldrich, #A9525 at 2 µg/g/d; diluted in 0.9% saline/0.01 M acetic acid) continuously for 28 days were implanted subcutaneously (s.c.) in the left flank of mice as previously described [50,61]. Initial anesthesia was achieved by intraperitoneal (i.p.) injection of a combination of medetomidine (500 µg/kg), midazolam (5 mg/kg), and fentanyl (50 µg/kg) and maintained throughout the surgical intervention with continuous inhalation anesthesia of 1.0–2.0 Vol% isoflurane/oxygen. At the end of the surgery, mice received flumazenil (0.5 mg/kg) and atipamezole (2.5 mg/kg) via s.c. application to antagonize anesthesia. Animal analgesia was intraoperative provided by administration of fentanyl i.p., followed by postoperative treatment with oral metamizole (1.25 mg/ml drinking water). Consistent with Kaur et al. (2016), Cre recombinase was activated by i.p. administration of 1 mg TAM (Sigma-Aldrich #T5648; dissolved in 50 µl Miglyol^®^812, Caelo #3274) once daily, starting on the first postoperative day, for five consecutive days [50]. Animals received only a daily injection of TAM for five consecutive days in the corresponding control experiments without Ang II. The examination of the hearts was performed 28 days after the first TAM injection.

### Telemetric BP measurements

To assess changes in BP, heart rate and locomotory activity in CTR and *cmf*KO mice as a result of prolonged Ang II infusion, implantable transmitters (Data Science International TA11PA-C10) allowing data acquisition by a telemetry system (Data Science International-Dataquest A.R.T.3.1 software), were employed described as previously described [61,62]. Briefly, anesthesia was induced by i.p. injection of a combination of medetomidine (500 µg/kg), midazolam (5 mg/kg), and fentanyl (50 µg/kg) and was supplemented after 30 min by continuous inhalation anesthesia with 1.0–2.0 Vol% isoflurane/oxygen. After the mice were placed in supine position on a heating plate to maintain body temperature at 37°C throughout the procedure, a ventral midline incision was made to allow the fluid-filled catheter to be inserted through a small incision into the left common carotid artery and advanced toward the thoracic aorta. The catheter was secured in this position with two additional sutures, with the transmitter unit placed subcutaneously on the right flank of the mice. At the end of the surgery, anesthesia was antagonized by flumazenil (0.5 mg/kg) and atipamezole (2.5 mg/kg) s.c. and analgesia was provided by perioperative administration of buprenorphine (0.05–0.01 mg/kg) s.c and by postoperative treatment with paracetamol p.o. (1.3 mg/ml drinking water). Following a seven-day recovery period, basal BP values were recorded at 15 min intervals for a duration of 5 min for three consecutive days. Immediately after implantation of Ang II releasing osmotic minipumps, BP was monitored at the previously indicated intervals for the following seven days.

### Transthoracic echocardiography

Non-invasive echocardiography was conducted using a Vevo2100 imaging system (FUJIFILM Visual Sonics) in CTRs and *cmf*KOs treated either with or without Ang II at day 28, as previously described [46,61,63]. In brief, animals were anesthetized by continuous inhalation anesthesia with 1.0–2.0 Vol% isoflurane/oxygen, restrained in supine position on a heating plate to maintain a body temperature of 37°C and depilated in the left thoracic region to avoid ultrasound artifacts. Limb electrodes permitted continuous ECG monitoring. Following the application of the ultrasound gel, high-resolution two-dimensional echocardiographic images were acquired using a 30-MHz transducer in the left parasternal long-axis view (PLAX). M-Mode recordings were acquired from three distinct selected positions for quantification of three cardiac cycles, respectively, allowing the determination of global cardiac function parameters such as ejection fraction (EF) and fractional shortening (FS), as well as wall dimensions from the mean of nine cardiac cycles.

B-Mode recording in PLAX enabled the evaluation of both further cardiac function parameters and, in particular, regional left ventricle deformation capacities using speckle-tracking echocardiography (STE) [64]. After acquisition of left ventricular (LV) wall motion by semiautomatic tracing of the endocardium and epicardium, a total of three consecutive cardiac cycles were evaluated and averaged for the evaluation of the global longitudinal strain (GLS). For the assessment of regional deformation capacity, the algorithm of the software (Vevo^®^ LAB, Vevo^®^ Strain Analysis) subdivided the LV along the long axis into six distinct regions (AB: anterior base, AM: anterior mid, AA: anterior apex, PB: posterior base, PM: posterior mid, PA: posterior apex). Strain [%], strain rate [1 /s], and velocity [cm/s] in LV longitudinal motion direction were obtained for each segment by LV wall motion tracing. Besides the peak values for each segment, the average strain values were additionally determined from the average curve generated by software. Three consecutive cardiac cycles were selected and averaged for analysis.

### Isolation of murine CF/CMF

For the isolation of CF/CMF primary cell cultures, CTR and *cmf*KO hearts obtained from Ang II-treated mice were harvested as quickly as possible and placed in ice-cold Dulbecco's Phosphate-Buffered Saline (DPBS). The atria along with the aorta were removed by a transversal incision through the heart, and ventricles were cleared of blood debris using DPBS, and then dissected into ~1 mm pieces. To extract the cells, pieces were digested for 80 min at 37°C in a water bath using a collagenase type II-based solution (ddH_2_O containing 85 mM Na-Glutamate, 60 mM NaCl, 10 mM HEPES, 5.6 mM KCl, and 1 mM MgCl_2_.6H_2_O supplemented with 1 mg/ml collagenase type II and 1 mg/ml bovine serum albumin, pH 7.4). In order to support this process and to preserve cell viability, the supernatant consisting of the digestion solution containing cells detached from the tissue was transferred to 26 ml of culture medium (DMEM+GlutaMAX^TM^ supplemented with 1% Penicillin Streptomycin solution (PenStrep), 10% Fetal bovine serum, and 1% Insulin–Transferrin–Selen) every 20 min to protect them from further digestion, while fresh digestion solution was added to the not yet completely dissolved heart pieces. Using a 40-µm cell strainer (Greiner bio-one #542040), as well as a centrifugation step (5 min at 300 rpm), non-digested heart segments and isolated CMs were separated from the CF/CMF cell fraction. Purification of CFs/CMFs was achieved by a further centrifugation step for 7 min at 1400 rpm. The obtained cell pellet was resuspended in 1 ml of culture media, counted using a Neubauer haemocytometer (Millipore #MDH-2N1) and plated out accordingly to the required cell number for each experiment.

### Statistical analysis

The GraphPad Prism 9.4.1 software was employed for the statistical analysis. All data are presented as mean + standard error of mean (SEM). Normal Gaussian distribution was confirmed by Shapiro–Wilk or Kolmogorov–Smirnov test. For the comparison of two groups (CTR *versus cmf*KO), the unpaired student *t*-test (*α* = 0.05) was performed in the presence of Gaussian normal distribution, and the Mann–Whitney *U* test was used as non-parametric test. In case of more than two groups (CTR+TAM; CTR+TAM+Ang II; *cmf*KO+TAM; *cmf*KO+TAM+Ang II), either the two-way ANOVA followed by a Šídák’s or Tukey’s multiple comparison test was performed or a Kruskal–Wallis test followed by the Dunn test for multiple comparisons was employed as non-parametric test. The log-rank test (Mantel-Cox test) was used to assess the survival rate. A more precise description of the statistical analyses conducted for the respective data sets is provided in the figure legends. *P*<0.05 were considered statistically significant with *P<0.05, **P<0.01, and ***P<0.001, indicating differences between the genotypes (CTR *versus cmf*KO). Comparison of the distinct treatments (+TAM *versus* +TAM+Ang II) within the same genotype was represented as followed: †*P*<0.05; §*P*<0.01; #*P*<0.001.

For all *in vivo* experiments, group sizes were equal by design, and G*Power (version 3.1.5) was used to calculate the required size of each experimental group of mice *a priori* with a power of 80% and an α level of 0.05. Randomization of the animals into different treatment groups was carried out before minipump implantation by generating random numbers for each individual animal. The different test conditions and experimental units were always carried out in parallel and with both animal genotypes at the same time. Genotype and treatment of the mice were not known to the experimenter and data analyst, so that an unbiased evaluation of the results was possible.

Statistics for main figures are listed together with raw data in Supplementary Table S1-S4.

## Results

### Validation of the cell-specific *Postn*iCre-mediated recombinase activity

To investigate the putative function of CMF-specific cGKI in Ang II-induced cardiac remodeling, we utilized a transgenic *Postn*iCre (genotype: *Postn*i*Cre* ^tg/+^) mouse line expressing a TAM-inducible Cre-recombinase under the control of the *Postn* promoter in CMFs [50]. First, the cell specificity and inducibility of *Postn*iCre activity were verified under patho-/physiological conditions. Therefore, *Postn*i*Cre* ^tg/+^ mice were intercrossed with a double-fluorescent ROSA^mT/mG^ Cre reporter mouse strain expressing membrane-targeted tandem dimer Tomato (mT) prior to Cre-mediated excision and membrane-targeted green fluorescent protein (mG) after excision of the loxP flanked mT cassette [59]. In unchallenged adult double-transgenic ROSA^mT/mG^ × *Postn*i*Cre* ^tg/+^ as well as ROSA^mT/mG^ mice used as controls, Cre-mediated recombination was induced by daily i.p. TAM-injection for five consecutive days (Supplementary Figure S1A). Four weeks after the first TAM injection, ubiquitous expression of the cell membrane-targeted mT protein was detected in the myocardium (Supplementary Figure S1B), as well as in multiple other organs including lung, aorta, liver, spleen, and kidney (Supplementary Figure S1C) of both genotypes. Importantly, we found no evidence of Cre-mediated excision of the mT transgene, which would lead to mG expression, confirming previous reports suggesting that *Postn^+^* CMF develops only under pathophysiological conditions [8,13,50]. Thus, we next analyzed *Postn*i*Cre*-mediated recombinase activity in the ROSA^mT/mG^ reporter strain in response to chronic Ang II released from osmotic minipumps. As Ang II-induced mRNA and protein expression of periostin is stimulated within hours in CFs [65], Cre-activation was induced by TAM on the first day after minipump implantation for five consecutive days (Supplementary Figure S2A). This previously established protocol [50] resulted in *Postn*i*Cre*-mediated excision of the loxP-flanked DNA sequence encoding mT, which was evident due to mG expression exclusively in cells occurring in fibrotic heart areas of double transgenic ROSA^mT/mG^ x *Postn*iCre^tg/+^ mice (Supplementary Figure S2B). Except for small fractions of cells in the lungs of double-transgenic mice, mG expression was restricted to myocardial CMFs and not detectable in CMs, as well as in non-fibrotic cardiac regions or any other organ systems analyzed including liver, spleen, kidney, and aorta, all of which exhibited persistent expression of the red fluorescent mT-protein (Supplementary Figure S2B and C). In order to confirm these findings at a cellular level, we isolated primary CFs/CMFs from ROSA^mT/mG^ × *Postn*iCre^tg/+^ and ROSA^mT/mG^ mice after Ang II treatment. In contrast with ROSA^mT/mG^ CF/CMF, which exclusively expressed the red fluorescent mT-protein, we obtained a mixed culture of primary CFs/CMFs from the double-transgenic hearts. These primary cultures consisted of cells stimulated *in vivo* by Ang II to form *Postn* ^+^ CMFs, characterized by the expression of the green fluorescent mG protein, and mT-positive CFs/CMFs (Supplementary Figure S2D). Due to the highly reliable TAM-controlled activation of the CMF-specific Cre-recombinase in Ang II exposed hearts, we conclude that the *Postn*iCre^tg/+^ mouse strain is suitable to assess the *in vivo* functions of cGKI in *Postn* ^+^ CMFs in adverse cardiac remodeling.

### Characterization of conditional CMF-specific cGKI KO mice

**Figure 1:**
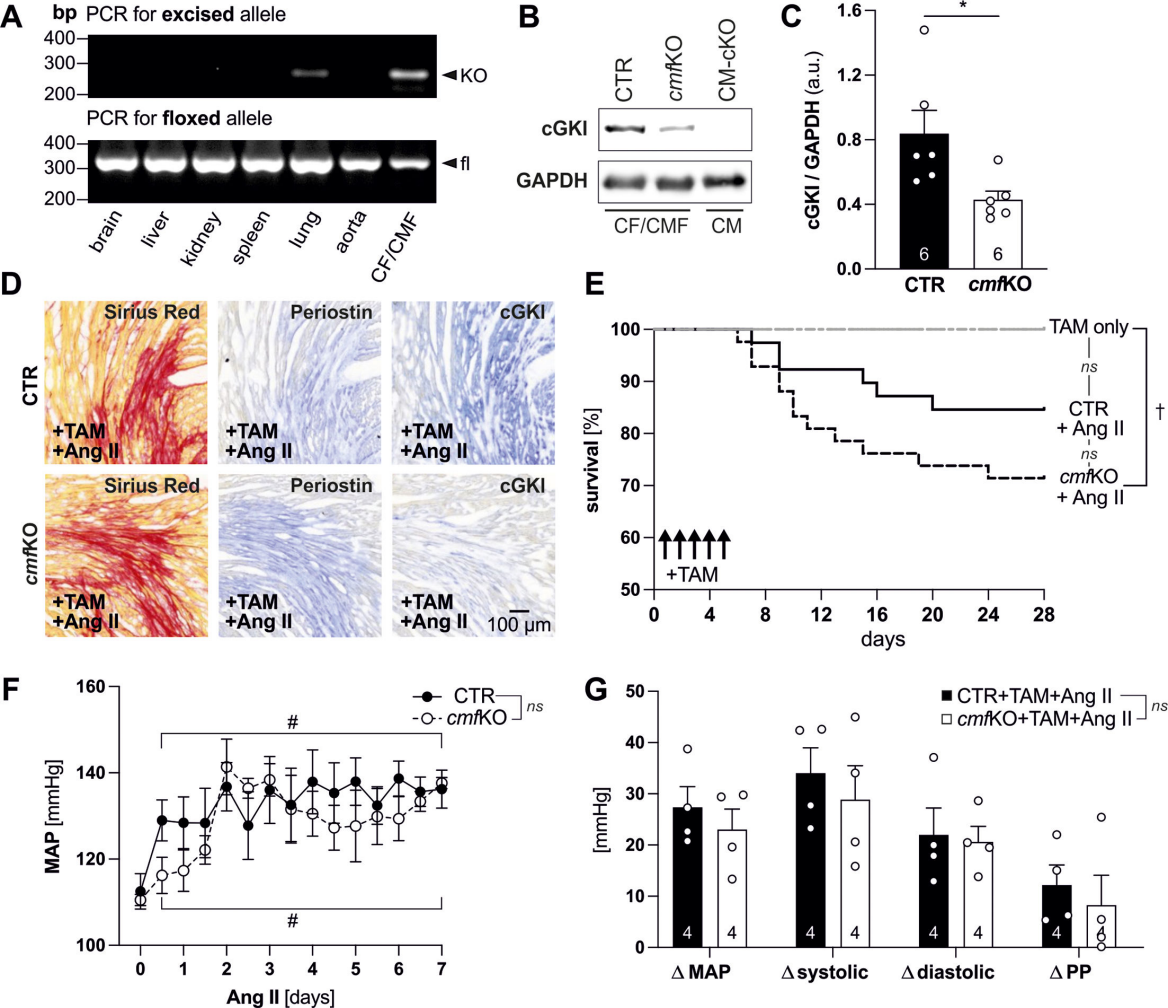
CMF-specific cGKI-KO mice exhibit a high vulnerability to chronic Ang II exposure. (**A**) Genomic PCR analysis demonstrates TAM-mediated cell-specific *Postn*iCre-recombination. DNA isolated from indicated tissues as well as primary CF/CMF of Ang II-treated *cmf*KO mice was amplified by allele-specific primer sets for *Prkg1*, encoding murine cGKI. As expected, conversion of the floxed *Prkg1* allele ([fl]; 338 bp) to the KO allele ([-]; 250 bp) was almost exclusively detectable in samples obtained from primary CF/CMF cultures, while recombination in the lungs did occur, but at a much lower level. Prior (data not shown) or 28 days upon TAM-induced *Postn*iCre recombination all other tissues tested remained negative for the KO-specific PCR product. (**B, C**) Representative immunoblot images of primary CF/CMF protein lysates obtained from +TAM and +Ang II-treated CTR mice exhibited a single band, corresponding to the expected molecular weight of cGKI, whereas cGKI immunoreactivity was largely attenuated in CF/CMFs derived from primary *cmf*KO cultures. GAPDH was utilized as loading control. CM protein lysates obtained from CM-specific cGKI KO (αMHC-Cre^tg/+^ x cGKI^fl/fl^; CM-cKO) hearts served as negative controls. (**C**) Quantification of the immunoblot shown in (**C**) with *N* = 6 protein samples per genotype. (**D**) Representative histochemical staining’s of consecutive heart cryosections derived from +TAM-treated CTR and *cmf*KO mice following 28 days of Ang II treatment. Fibrotic areas were visualized by PSR staining (left panels). Validated antibodies specifically targeting periostin (middle panels) [66] or cGKI (right panels) [67] were used to detect the respective proteins in serial sections adjacent to histologically detectable myocardial fibrosis. While periostin was restricted to the fibrotic cardiac areas in both genotypes, cGKI expression in *cmf*KO *versus* CTR hearts was largely depleted within the PSR- and periostin-positive heart area. In total, *n* = 3 cryosections were evaluated per heart deriving from *N* = 3 mice per genotype. Scale bar = 100 µm. (**E**) Kaplan–Meier analysis of CTR and *cmf*KO mice following either +TAM (CTR, *N* = 13; *cmf*KO, *N* = 12) or +TAM and +Ang II (CTR, *N* = 39; *cmf*KO, *N* = 42) treatment. Overall survival within +TAM-treated groups was not affected by genotype (*n.s*.), and all mice survived the respective treatment. With chronic Ang II exposure, 85% of CTR mice survived the indicated treatment, whereas the survival of *cmf*KO mice was 71% and therefore significantly lower compared with the corresponding TAM-treated *cmf*KO group. (**F**) Time course of MAP changes in response to Ang II during the first seven days (day = 1–7) after osmotic pump implantation (day = 1) yielded no genotype-related differences although, as expected, the Ang II treatment by itself significantly increased BP compared with the average MAP values obtained during the last 24 h of the basal measurements, i.e., immediately prior to minipump implantation (day = 0). *N* = 4 unrestrained and awake mice per genotype carrying telemetry implants were monitored prior and during the first seven days of the Ang II infusion. (**G**) Quantification of the relative increase (compared with basal values) in MAP, systolic and diastolic BP, and pulse pressure (PP) expressed as △mmHg exhibited no genotype-related differences. **Statistics**: (**C, F, and G**) Data are represented as mean + or ± SEM with *P<0.05 (**C**) using an unpaired *t*-test and (**E**) the log-rank test (Mantel-Cox test) to assess the survival rate with †*P*<0.05 revealing differences between the distinct treatments (+TAM *versus* +TAM+Ang II) within the *cmf*KO group. (**F**) Two-way ANOVA followed by Šídák’s multiple comparisons test showed a significant increase (#*P*<0.001) in BP over time due to the +TAM+Ang II treatment within both the CTR and *cmf*KO group, but no differences between genotypes (*n.s*.). (**G**) Multiple unpaired *t*-test corrected for multiple comparison using the Holm-Šídák method revealed again no differences (*n.s*.) on all parameters plotted between the genotypes due to the +TAM+Ang II treatment. Further details concerning statistics are listed together with raw data in Supplementary Table S1. Ang II, angiotensin II; CF, cardiac fibroblast; CMF, cardiac myofibroblast; CTR, control; MAP, mean arterial pressure; PP, pulse pressure; TAM, tamoxifen.

CMF-specific KO mice (*cmf*KO; genotype: *Postn*iCre^tg/+^ x cGKI^fl/fl^) lacking exon 10 of the cGKI gene required for proper kinase activity, as well as respective control mice (CTR; genotype: *PostniCre* ^tg/+^ x cGKI^+/+^) (Supplementary Figure S3A), were subjected to the herein established TAM and Ang II treatment protocol (Supplementary Figure S2A). Consistent with the previous findings (Supplementary Figure S2B-D), genomic PCR analysis confirmed efficient conversion of the floxed *Prkg1* allele into the KO allele in both primary CF/CMF cultures and, to a lesser extent, in the lungs obtained from Ang II-treated *cmf*KO mice (Figure 1A). Neither in other organs isolated from Ang II-treated *cmf*KO nor in the DNA samples obtained from the corresponding Ang II-treated CTR mice, we observed a recombination of the floxed cGKI gene locus (Figure 1A, Supplementary Figure S3B). Furthermore, immunoblots using a validated cGKI antibody [67] exhibited significantly reduced cGKI protein levels in primary CFs/CMFs obtained from Ang II-treated *cmf*KO animals compared with corresponding Ang II-treated CTR mice (Figure 1B and C). CMs isolated from an alternative and highly efficient CM-restricted cGKI-KO model (CM-cKO; genotype: αMHC-Cre^tg/+^ x cGKI^fl/fl^) used as negative control confirmed the high specificity of this approach. Next, we evaluated the cGKI expression pattern in CTR and *cmf*KO heart sections obtained from Ang II-treated mice. In CTR sections, cGKI expression correlated with the fibrotic myocardium and with *Postn* ^+^ cells, while *cmf*KO hearts exhibited a substantial reduction in cGKI expression in myofibroblast-rich cardiac sections (Figure 1D).

### Survival and BP analysis of Ang II-treated *cmf*KO and CTR mice

Depletion of cGKI in CMF resulted in a significantly reduced survival of TAM-treated *cmf*KO mice during the Ang II challenge, whereas TAM in the absence of Ang II did not affect the overall survival of age- and littermate-matched conditional mutants (Figure 1E). Although TAM in combination with Ang II slightly decreased the survival of CTR animals, this trend was not significantly different from either the *cmf*KO treatment group or the CTR animals receiving TAM only (Figure 1E). With regard to this excess in mortality in both +TAM+Ang II treatment groups, we identified perivascular and also tubulointerstitial fibrosis in the kidney but no genotype-related adverse remodeling events (data not shown). Accordingly, the integrity of the elastic fibers as well as the thickness of the muscular layer of the thoracic and abdominal aorta showed neither massive abnormalities nor differences between +TAM+Ang II-treated CTR and *cmf*KO mice (data not shown), although reportedly the architecture of the vessel wall can be disrupted by the development of aortic aneurysms in response to Ang II [68].We next monitored the BP of CTR and *cmf*KO mice because Ang II causes hypertension through different mechanisms, whereas the contribution of the cGMP/cGKI signaling pathway in CMF to BP control is less clear. Prior to TAM-induced Cre recombination and the Ang II treatment, neither mean arterial pressure (MAP) nor systolic and diastolic BP were statistically different between the two genotypes. This was accompanied by nearly identical pulse pressure (PP) and heart rate (HR) values as well as similar activity patterns of he mice throughout the day and night (Supplementary Figure S4A-F). Infusion of Ang II resulted in a significant and time-dependent elevation of the MAP in CTR and *cmf*KO mice; however, the extent of this increase was identical in both genotypes (Figure 1F). Also, HR, systolic and diastolic BP, as well as PP elevations, and locomotor activity were identical for CTR and *cmf*KO mice receiving Ang II (Supplementary Figure S4G-L). Thus, a genotype-related difference in the absolute response of MAP, systolic BP, diastolic BP, and PP, the latter being an important predictor of cardiac complications [69,70], to chronic Ang II exposure could be excluded (Figure 1G). Combined, these results strongly suggest that the poor survival and higher myocardial vulnerability of *cmf*KO may be due to local effects of the neurohormone on the myocardium, rather than Ang II-related changes in systemic BP control.

### CMF-specific cGKI provides protection against Ang II-induced cardiac remodeling

**Figure 2:**
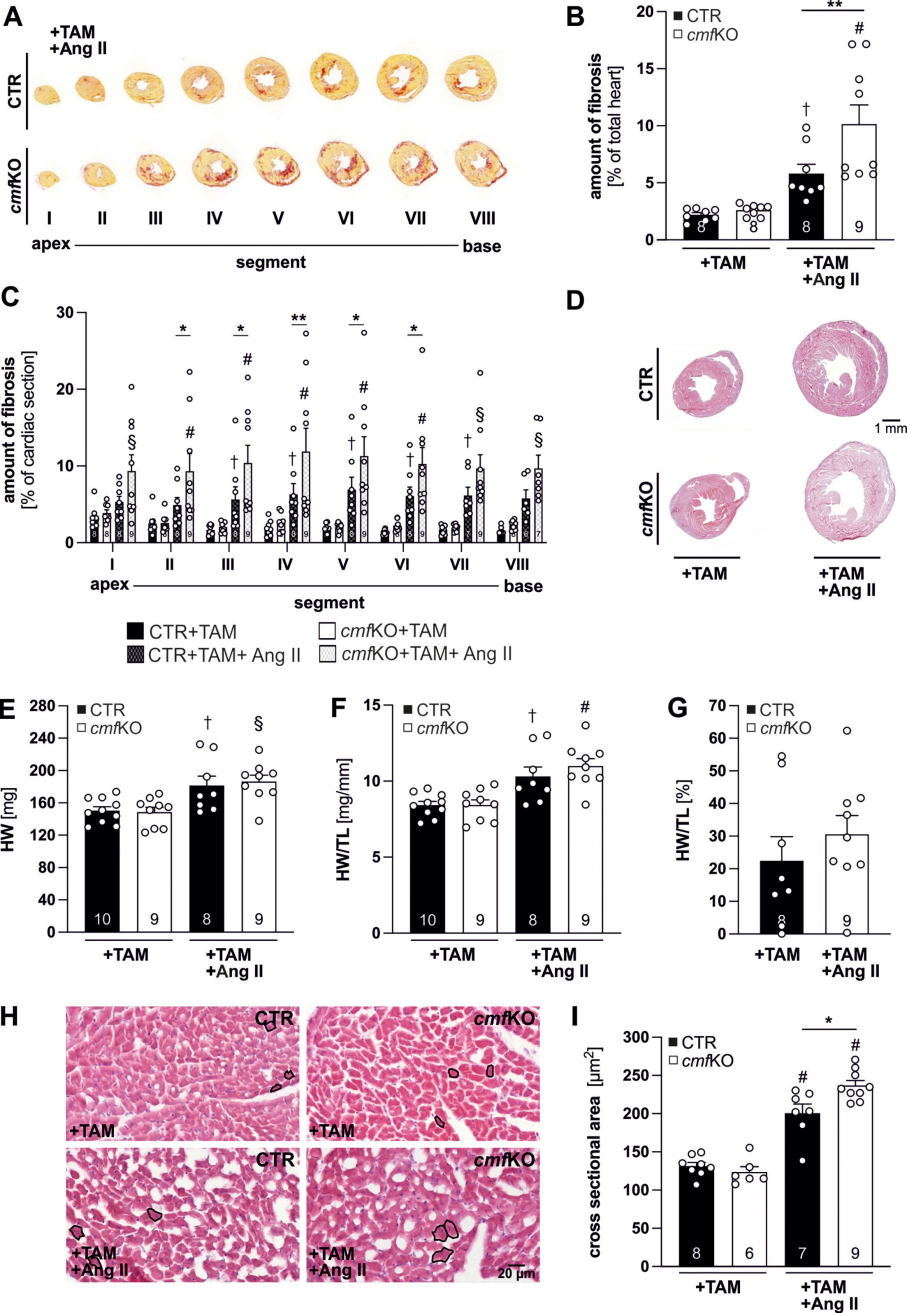
Ang II provokes greater histopathological changes in cmfKO hearts. (**A**) Hearts derived from +TAM and +TAM plus Ang II-treated CTR and *cmf*KO animals were divided into eight equidistant cardiac segments, ranging from the apex (segment I) to the base of the hearts (segment VIII) and were subsequently stained with PSR to (**B**) quantify the amount of fibrosis as percentage of the whole myocardial area within each segment. These analyses revealed an overall significantly higher percentage of fibrosis in *cmf*KO hearts (*N* = 9) compared with CTR hearts (*N* = 8), which was due to an elevated (**C**) amount of collagen depositions in each (I–VIII) of the *cmf*KO heart segments. Compared with the +TAM-treated groups the +TAM+Ang II treatment exaggerated fibrosis in segments II–VI of the cmfKO compared with CTR. In (**C**) *n* = 2–4 consecutive heart slices per segment (I–VIII) per animal (*N* = 9 *cmf*KO and *N* = 8 CTR) were included in the analysis. (**D**) Representative H&E staining of whole heart cross-sections obtained from +TAM or +TAM+Ang II-challenged CTR and *cmf*KO mice. Both genotypes showed pronounced hypertrophy development to a comparable extent upon the chronic Ang II exposure (CTR, *N* = 8; *cmf*KO, *N* = 9), as indicated by significant increases in (**E**) total HW, (**F**) HW/TL ratio as well as (**G**) in the percentage increase in HW/TL compared with the corresponding +TAM control groups (CTR, *N* = 10; *cmf*KO, *N* = 9). (**H**) Representative H&E-staining’s of cardiac cryosections from CTR and *cmf*KO after either +TAM or +TAM+Ang II treatment. (**I**) CM cross-sectional areas significantly increased in both genotypes after Ang II infusion (CTR, *N* = 7; *cmf*KO, *N* = 9) compared with their respective control (+TAM) group (CTR, *N* = 8; *cmf*KO, *N* = 6). Ang II-induced hypertrophic CM growth, however, resulted in a greater enlargement in *cmf*KO compared with CTR hearts. Cross-sectional areas of *n* = 150 CMs were evaluated per individual heart. Representative cells that were included in the statistics are outlined in black in all panels. **Statistics**: (**B**) Two-way ANOVA followed by the two-stage linear step-up procedure of Benjamini, Krieger, and Yekutieli, with statistical significance determined by a false discovery rate (FDR) threshold of *q*<0.05. Significant differences were found at **P<0.01 between genotypes and at †*P*<0.05 or #*P*<0.001 between distinct treatments (+TAM *versus* +TAM+Ang II) within the same genotype as indicated. (**C**) Two-way ANOVA followed by the two-stage linear step-up procedure of Benjamini, Krieger, and Yekutieli, with statistical significance determined by a FDR threshold of *q*<0.05. Significant differences were found at *P<0.05 and **P<0.01 between genotypes in the respective segments and at †*P*<0.05, §*P*<0.01, and #*P*<0.001 for distinct treatments (+TAM *versus* +TAM+Ang II) within the same segment/genotype. (**E**, **F**, and **I**). Two-way ANOVA followed by Tukey’s multiple comparisons test revealing significant differences at *P<0.05 between genotypes and at †*P*<0.05, §*P*<0.01, or #*P*<0.001 between the distinct treatments (+TAM *versus* +TAM+Ang II) within the same genotype. (**G**) Unpaired *t*-test did not show a difference between the [%] increase in HW/TL between genotypes. Details concerning statistics are listed together with raw data in Supplementary Table S2. Ang II, angiotensin II; CF, cardiac fibroblast; CMF, cardiac myofibroblast; CTR, control; HW, heart weight; TAM, tamoxifen; TL, tibia length.

Based on the assumption that Ang II directly and independent of BP elevation promotes cardiac remodeling, we next assessed the extent of myocardial fibrosis and hypertrophy in CTR, as well as *cmf*KO hearts following the TAM protocol and a sustained Ang II treatment. Untreated CTR and *cmf*KO mice were also included in this analysis to exclude genotype-specific differences in the histological properties of the heart under physiological conditions. Again, all mice received +TAM to induce *Postn*iCre-mediated recombination in CMFs. For the evaluation of fibrosis development, hearts were divided into eight equidistant regions, proceeding from the apex to the base of the heart. Thereby, picrosirius red (PSR) staining, highlighting collagen fibers in the connective tissue, identified a significant increase in global amount of cardiac fibrosis in *cmf*KO compared with corresponding CTR mice (Figure 2A and B). This increase was seen in each individual cardiac segment obtained from *cmf*KO hearts (Figure 2C) and reached significance in segments II–VI also for the comparison with the CTR. Accordingly, the loss of cGKI in CMF was associated with a higher type I and III collagen content of myocardial segments IV–VI (Supplementary Figure S5A-D), which is a significant finding, as these two collagens are the main isoforms of this ECM protein that accumulate in response to Ang II [14,71,72]. In contrast, our parallel examination of cardiac sections obtained from TAM but not Ang II challenged mice yielded no genotype-specific differences in the PSR-stained collagen fiber content (Figure 2B and C).

Prolonged Ang II treatment resulted in pathological cardiac enlargement in both genotypes. However, heart weight (HW) and the heart weight-to-tibia length (HW/TL) ratio, considered a surrogate parameter for the development of cardiac hypertrophy [73], were not different between *cmf*KO and respective CTR mice. Accordingly, the amount of cardiac mass gained did not differ between both genotypes, as further confirmed by the percentage increase in HW/TL ratio. Compared with the respective TAM-treated animals, however, both HW and HW/TL ratios of Ang II-treated mice were significantly higher for both genotypes, which again confirms the validity of the methodology and protocols used (Figure 2E–G). Because CM hypertrophy, in addition to changes in ECM composition, is responsible for the abnormal increase in myocardial mass, we determined CM sizes in hematoxylin and eosin (H&E) stained heart slices from untreated or Ang II-treated CTR and *cmf*KO mice [74]. Compatible with the cardiac enlargement and mass gain, we observed a significant increase in CM cross-sectional areas in both Ang II-treated genotypes as compared with their respective control groups that received TAM only. Consistent with our data quantifying fibrosis, this increase was more pronounced in Ang II-treated *cmf*KO *versus* CTR mice (Figure 2H and I). In a complementary approach, we used fluorescently labeled wheat germ agglutinin (WGA) to mark the borders of the CMs (Supplementary Figure S5E). Again, cross-sectional areas of randomly chosen cells were determined. Although the absolute values of the randomly selected cells differed slightly from those determined by examining the H&E-stained heart sections, significant size differences between the two genotypes were found only after +TAM+Ang II exposure (Supplementary Figure S5F).

**Figure 3:**
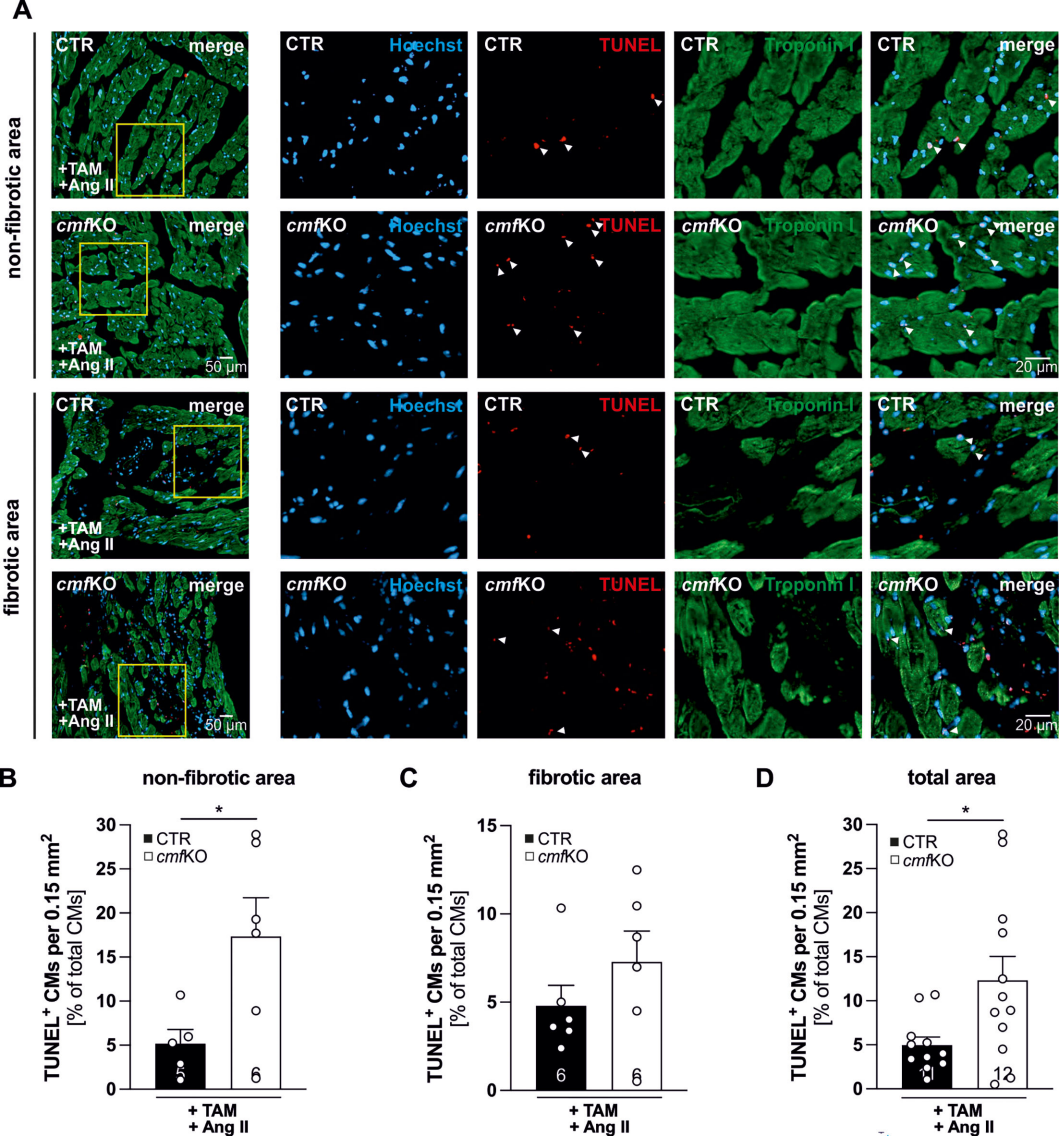
CMF-specific ablation of cGKI resulted in higher CMs cell death after Ang II infusion. Assessment of CM cell death in heart slices obtained from +TAM- and Ang II-treated CTR and *cmf*KO animals. (**A**) Representative images of the TUNEL assay co-stained with troponin I for identification of CMs in both fibrotic and non-fibrotic cardiac regions. Quantification of TUNEL-positive CMs, expressed as a percentage of the total number of CMs, revealed a significantly increased cell death rate in (**B**) non-fibrotic cardiac regions of the *cmf*KO hearts (*N* = 6) in response to prolonged Ang II stimulation compared with the corresponding CTR hearts (*N* = 5). (**C**) In contrast, no genotype-related difference could be detected within fibrotic cardiac regions (*N* = 6 for each genotype) containing proliferating non-CM. (**D**) If the two previously separately analyzed heart areas are considered together, higher CM cell death was confirmed in *cmf*KO (*N* = 12) compared with CTR hearts (*N* = 11). For each animal, *n* = 3 non-fibrotic/fibrotic areas, originating from two different heart segments (IV, VI), within n = 6 heart slices comprising an area of 0.15 mm^2^ were analyzed. **Statistics**: Data in (**B**) and (**D**) are represented as mean  +  SEM with *P<0.05 indicating significant difference between genotypes (unpaired student *t*-test). Additional information on statistics are presented together with raw data in Supplementary Table S3. Ang II, angiotensin II; CF, cardiac fibroblast; CMF, cardiac myofibroblast; CTR, control; TAM, tamoxifen.

To clarify the apparent discrepancy regarding the massive increase in fibrosis (Figure 2A and B) and the enlarged CMs in the presence of identical heart weights compared with the controls (Figure 2D–G), we quantified CM cell viability by TUNEL staining (Figure 3). Because the number of apoptotic CMs was significantly increased in CMF-specific cGKI KO hearts, we conclude that a functional cGMP/cGKI cascade in CMF improves the survival of CMs in the presence of Ang II. In addition, the higher cell death rate in the *cmf*KO explains why the total weight of the heart, despite the greater fibrosis of the cardiac muscle and the larger CMs, does not differ between conditional cGKI mutant and CTR mice. Next, we investigated whether these unique alterations in CM size, collagen density, and total organ weight were caused by an unexpected change in the cardiac periostin expression pattern. In the absence of Ang II and fibrotic lesions, we were unable to detect periostin in cardiac section of either genotype (Supplementary Figure S5G), while transcript levels (Supplementary Figure S6A and B) and the number of periostin-positive primary CF/CMFs obtained from +TAM+Ang II treated *cmf*KO and CTR mice were identical (Supplementary Figure S6C). This indicates that the matricellular protein periostin, which may actively contribute to tissue fibrosis and remodeling processes, is not responsible for the *in vivo* phenotype we observed in the absence of cGKI in CMFs.

### Analysis of proliferation rates, CF/CMF transcript expression, and cGKI activity in primary cells obtained from Ang II-treated *cmf*KO hearts

**Figure 4:**
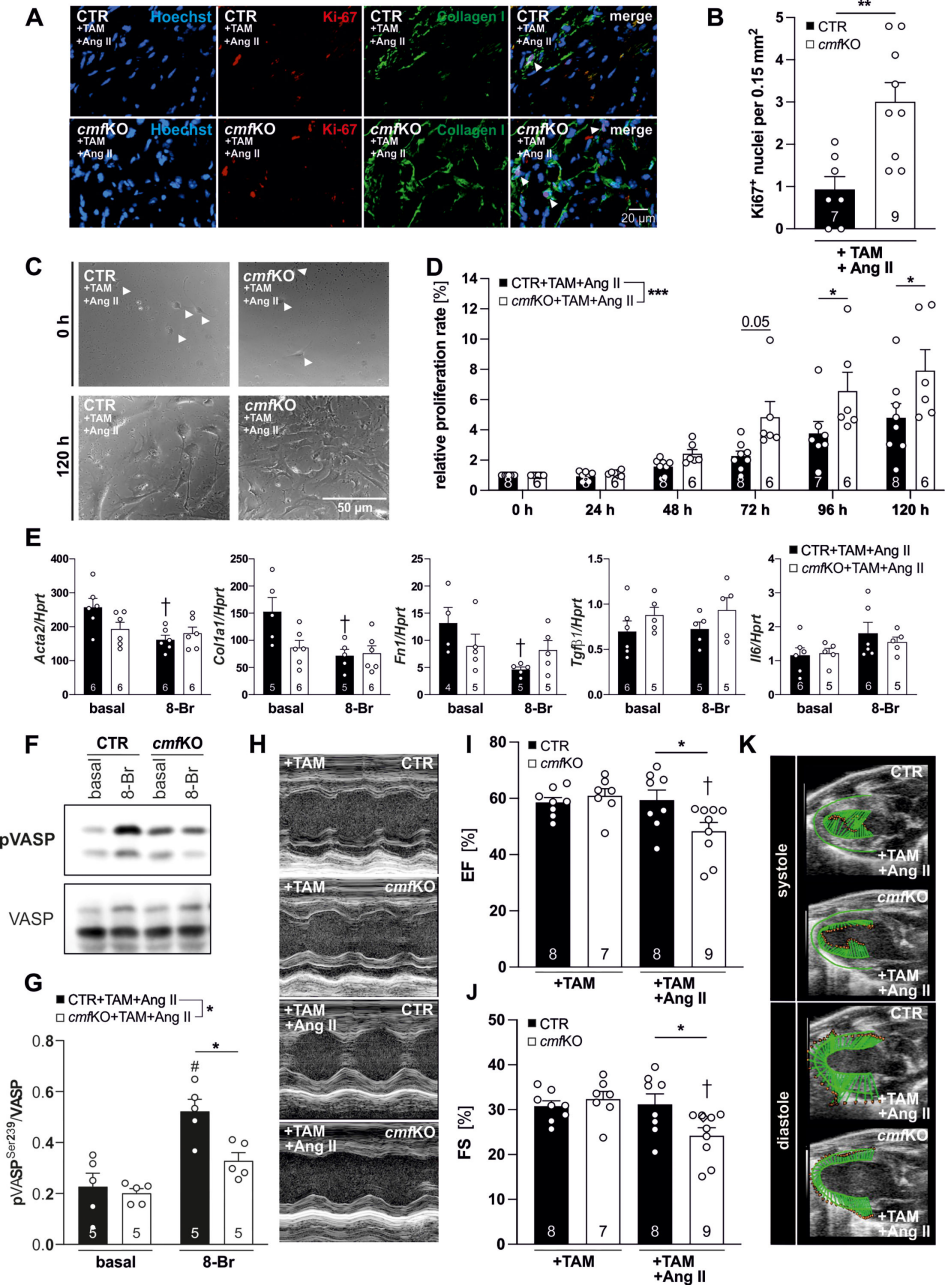
Loss of CMF-specific cGKI causes CF/CMF hyperproliferation, relieves anti-fibrotic effects on gene transcription by 8-Br-cGMP *in vitro,* and results in a worsening of LV function *in vivo*. (**A**) Immunofluorescence staining and (**B**) quantification of the proliferation marker Ki-67. Collagen I antibodies were used in order to localize the fibrotic area containing CMFs. To calculate the proliferative index, the number of Ki-67^+^ cells was related to defined areas of fibrosis. Analysis of *n* = 3 areas comprising 0.15 mm^2^ in distinct heart segments (segment IV–VI) exhibited a significantly increased accumulation of Ki-67^+^ nuclei in *cmf*KO (*N* = 9) *versus* CTR (*N* = 7). (**C**) Representative images acquired from the grid-based proliferation assay. CF/CMF isolated from either CTR or *cmf*KO mice receiving +TAM+Ang II *in vivo* for 28 days are shown after *t* = 120 h in primary culture. (**D**) During the five-day monitoring period, this assay revealed a significantly enhanced proliferation rate of primary *cmf*KO (*N* = 6) *versus* CTR (*N* = 8) CF/CMF cells. *n* = 3 replicates were assessed per genotype per experiment. (**E**) qRT-PCR analysis of common fibroblast marker transcripts from +TAM and +Ang II-treated CTR and *cmf*KO hearts. Stimulation of CF/CMF with 8-Br cGMP (8-Br; 1 mM) for 24 h revealed that levels of *Acta2*, *Col1a1,* and *Fn1*, but not of *TGFβ*1 and *Il6*, were sensitive to the 8-Br cGMP treatment in CTR cells. In contrast, transcript levels of all pro-fibrotic markers examined did not differ for *cmf*KO both under basal and stimulated conditions. (**F**) CTR and *cmf*KO CF/CMF obtained from +TAM+Ang II *in vivo* treated mice (28 d) were stimulated in culture using 8-Br for 30 min to induce VASP phosphorylation at Ser239 (pVASP^S239^). (**F**) Representative immunoblots and (**G**) relative densitometric bar graphs with individual data points plotted revealed a significant increase in the pVASP^S239^ to VASP ratio only in the presence of cGKI, i.e., in CTR samples. (**H**) Representative M-mode images in the PSLAX view of CTR and *cmf*KO mice either after TAM injection alone or following additional 28 days of Ang II treatment. While CTR animals (*N* = 8) displayed no deterioration of global cardiac function after chronic Ang II exposure *versus* unchallenged CTR animals (*N* = 8), non-invasive analysis of the global heart function yielded a significant decline in (**I**) EF and (**J**) FS in *cmf*KO mice (*N* = 9) as compared with Ang II-treated CTR and corresponding TAM-treated *cmf*KO (*N* = 7) groups. (**K**) Regional analysis of wall motion tracking in PSLAX B-mode during systole and diastole is expressed by velocity vectors in representative images obtained from CTR and *cmf*KO mice subjected to 28 days of Ang II treatment. Both the systolic and the diastolic velocity vectors were shorter in *cmf*KO heart compared with CTR hearts, indicating an impairment of the ventricular wall motion. **Statistics**: Data in (**B**) are represented as mean  +  SEM with **P<0.01 using an unpaired student *t*-test. (**D**) Two-way ANOVA with ***P<0.001 (F_1,71_ = 16.47) showing significant difference for the overall proliferation rate between genotypes followed by Šídák’s multiple comparisons test revealing significant difference at *P<0.05 for 96 h and 120 h between genotypes. (**E**) Two-way ANOVA followed by Tukey’s multiple comparisons test with †*P*<0.05 indicating significant difference for the *Acta2/Hprt* (F_1.20_ = 7.3) *Col1a1/Hprt* (F_1.18_ = 7.540) and the *Fn1/Hprt* (F_1.15_ = 5.836) ratios between the distinct treatments (basal *versus* 8-Br) for the CTR+TAM+Ang II group. (**G**) Two-way ANOVA followed by Tukey’s multiple comparisons test with *P<0.05 (F_1.16_ = 7.63) showing significant difference for the pVASP/VASP ratio between genotypes (considering all basal and 8-Br values) and for the 8-Br condition (*P<0.05). Only the values in the CTR group reached the significance level at #*P*<0.001 for the comparison of the basal *versus* 8-Br condition. (**I, J**) Two-way ANOVA followed by Tukey’s multiple comparison test with significant difference *P<0.05 between genotypes and †*P*<0.05 revealing differences between the distinct treatments (+TAM *versus* +TAM+Ang II) within the *cmf*KO group. Further details are listed together with raw data in Supplementary Table S4. 8-Br, 8-Br cGMP; Ang II, angiotensin II; CF, cardiac fibroblast; CMF, cardiac myofibroblast; CTR, control; TAM, tamoxifen; VASP, vasodilator-stimulated phospho-protein.

To test whether the enhanced collagen deposition in *cmf*KO hearts was due to an accelerated proliferation rate of CMFs lacking cGKI [42,75,76], we visualized fibrotic areas by an anti-type I collagen immunofluorescence staining in Ang II-treated *cmf*KO and CTR hearts and used Ki-67 expression as marker for CMF proliferation. Thereby, we detected a significantly increased number of Ki-67-positive nuclei in *cmf*KO compared with CTR hearts, indicative of a higher cellular proliferation rate in the absence of CMF-specific cGKI (Figure 4A and B). To attribute the higher proliferation rate to impaired cGMP/cGKI signaling in *cmf*KO-derived CMFs, primary CFs/CMFs were isolated from TAM- and Ang II-treated CTR and *cmf*KO animals and cultured in a grid-based well system. The purity of cell cultures obtained was routinely verified by quantifying transcript levels of marker genes preferentially expressed either by CMs (*Tnni3*/cTNI) or endothelial cells (*Icam1*/ICAM-1) and by assessing *Pecam1*/PECAM-1, a broad cell surface marker of hematopoietic and immune cells including platelets (Supplementary Figure S6A and B). While these cell markers were expressed at very low levels, mRNA transcripts of periostin and α-SMA (*Acta2*), both representing widely used and specific markers of myofibroblast differentiation, were present at several orders of magnitude higher and, importantly, did not differ between the genotypes (Supplementary Figure S6B). This enabled us to monitor the growth of highly pure CF/CMF cultures containing >85% of periostin-positive cells (Supplementary Figure S6C and D) *in vitro* over a duration of five days (Supplementary Figure S6A). Consistent with the Ki-67-staining of the myocardium (Figure 4A and B), primary CF/CMF cultures obtained from +TAM and +Ang II *cmf*KO hearts exhibited significantly increased growth rates compared with the respective CTR cell cultures (Figure 4C and D), a finding that was further confirmed by counting the respective cell nuclei at the indicated time points (Supplementary Figure S6E and F).

By using the membrane-permeant cGMP-analogue 8-Br-cGMP on primary CF/CMF cultures, we next tested whether an activation of the cGMP/cGKI axis would also interfere with the production of pro- and/or anti-fibrotic factors in these cells. To address this, CF/CMF cultures were stimulated with 8-Br-cGMP for 24 h and transcript analysis was performed on total RNA isolated from CTR and *cmf*KO cells (Supplementary Figure S6A). 8-Br-cGMP, in a cGKI-dependent manner, interfered with the expression of pro-fibrotic marker genes such as *Acta2*, *Col1a1* and *Fn1*, whereas levels of *IL-6* and *TGFβ1* were not affected by 8-Br-cGMP/cGKI in cells obtained from +TAM- and +Ang II-treated CTR hearts. As we did not detect significant differences between genotypes, we conclude that the effects of cGKI on the pro-fibrotic gene expression profile contribute to a lesser extent than the effects of the pathway on the proliferative behavior of CF/CMF (Figure 4C and D and Supplementary Figure S6E) to the observed *in vivo* phenotype.

Finally, we assessed the phosphorylation of the vasodilator-stimulated phospho-protein (VASP) to demonstrate a proper activation of cGKI in response to the 8-Br-cGMP treatment. Indeed, 30 min of exposure increased the phospo-VASP (pVASP) to VASP ratio only in CTR cells, while pVASP and VASP levels, as well as the respective ratio, remained at the basal level in the absence of CMF cGKI (Figure 4F and G). This finding once again emphasizes a proper pharmacological modulation of cGKI in CTR *versus cmf*KO CMF.

### Structural and functional distortion of Ang II-treated *cmf*KO hearts

Cardiac fibrosis interferes with a proper cardiac contractility and relaxation eventually limiting cardiac function [2]. Consequently, we applied non-invasive echocardiography technique to investigate structure- and function-related cardiac dysfunctions of CTR and *cmf*KO hearts at baseline and upon *in vivo* Ang II exposure. Consistent with the histochemical alterations, we detected a deteriorated global cardiac function in *cmf*KO hearts, characterized by a significant reduction in the ejection fraction (%EF) and fractional shortening (%FS) (Figure 4H–J). The extent of dysfunction was significant compared with corresponding TAM-treated *cmf*KO, as well as Ang II-treated CTR animals. The latter even showed the ability to functionally compensate for the cardiac damage induced by Ang II as %EF and %FS remained unchanged compared with untreated control animals. Additionally, end-systolic and end-diastolic measurements of the intraventricular septum (IVS) and LV posterior wall (LVPW) were evaluated in order to provide detailed insights into LV chamber dimension, i.e., wall thickening and motion. LV mass estimation by echocardiography identified an increase in both genotypes, which reached a statistical significance only for the *cmf*KO compared with their respective TAM-treated control group (Supplementary Figure S7A). Quantification of wall dimensions revealed a significant thickening of both the end-systolic LVPW in CTR hearts and of the end-diastolic IVS and LVPW in CTR and *cmf*KO hearts (Supplementary Figure S7B-J). Furthermore, we found evidence for a disturbed wall motion in the LVPW segment of Ang II-treated *cmf*KO as end-systolic to end-diastolic wall diameters were significantly reduced only in this group of animals (Supplementary Figure S7J). To investigate this in more detail during the cardiac cycle, strain analyses based on STE were carried out. STE measures not only wall endpoints but also LV wall motion and deformability, providing a highly sensitive and validated quantitative measure of myocardial contractile function [64,77]. LV wall motion, here expressed by velocity vectors, was reduced, as indicated by the shortened length of the vectors, in Ang II-treated *cmf*KO compared with CTR mice in both systole and diastole (Figure 4K). Moreover, GLS, referring to myocardial shortening and lengthening of the entire LV in a longitudinal direction throughout a cardiac cycle [78], revealed a deterioration of the overall deformation capacity of *cmf*KO hearts due to the prolonged Ang II infusion (Supplementary Figure S8A). These findings prompted us to further investigate specific regional deformation capacity of the cardiac muscle within six distinct cardiac segments automatically separated by the VevoStrain2100 software (Supplementary Figure S8B). Assessment of the regional longitudinal peak strain, as well as longitudinal peak strain rate, with the latter providing a measure of the deformation per time [79], exhibited a significantly impaired endocardial longitudinal deformation capacity in almost all cardiac regions of Ang II-treated *cmf*KO hearts (Supplementary Figure S8B-F). Besides other segments especially in the AA region, myocardial deformation was strongly aggravated in the absence of CMF-specific cGKI.

In conclusion, consistent with the underlying morphological and structural changes in response to the Ang II-induced cardiac injury, *cmf*KO hearts developed a severe loss of cardiac function as reflected by a decrease in %EF and %FS and a regional dysregulation of LV motion.

### Discussion

CMFs are the major source of ECM-proteins such as fibronectin, type I, and type III collagens in the injured heart. While the majority of quiescent CFs are characterized by the expression of transcription factor 21 (TCF21), CMFs exhibit elevated levels of markers such as α-SMA and periostin, which are considered important profibrotic cues for myofibroblast activation [80]. Periostin*,* induced by Ang II in CF within hours [24,65]*,* represents a secreted matricellular protein enriched in collagen-rich connective tissue contributing to collagen fibrillogenesis and thus ECM re-/organization [81,82]. Accordingly, acquired *Postn* expression correlates highly with the activated CF phenotype and is postulated to be a common marker of CMFs, irrespective of their origin [13,50]. Although it has long been known that CMFs play a crucial role for the clinical course of HF, the specific analysis and manipulation of this cell type has been difficult due to the lack of specific cell markers [83]. By employing an innovative TAM-inducible Cre-recombinase under control of the *Postn*-promotor [50], we herein generated a CMF-restricted cGKI KO mouse model and investigated whether previously recognized antifibrotic properties attributed to cGMP/cGKI are due to the *in vivo* actions of this pathway in *Postn* ^+^ CMFs (Supplementary Figure S3A).

First, we verified the temporal, spatial, and cell type-specific control of the *Postn*iCre recombinase utilizing the ROSA^mT/mG^ Cre reporter strain [46,59]. We were unable to detect TAM-induced *Postn*-mediated recombination under physiological conditions, suggesting tight regulation of recombinase activity (Supplementary Figure S1). However, after chronic Ang II exposure and TAM-treatment for five days *in vivo* (Supplementary Figure S2), the excision of the loxP-flanked DNA sequence encoding mT resulted in mG expression in fibrotic but not in non-fibrotic heart regions. Furthermore, we were able to detect recombination, albeit to a much lesser extent, in the double-transgenic ROSA^mT/mG^ x *Postn*i*Cre* ^tg/+^ lungs, which is consistent with reports suggesting that *Postn* plays a key role in lung fibroblast function [84]. In line with these observations, lineage tracing analyses using an alternative TAM-inducible *Postn* mouse model based on MerCreMer, a Cre fusion protein containing two modified ER ligand binding domains, revealed *Postn* expression in less than 1% of interstitial cells in various healthy tissues [13]. Kanisicak et al. (2016) and others, in turn, showed that cardiac damage to different stresses results in an accumulation of *Postn* ^+^ cells, triggering muscle fibrosis [50]. Accordingly, targeted ablation of *Postn* ^+^ CMFs prevented the cardiac remodeling in response to Ang II and after MI [50]. Thus, the herein observed *Postn*iCre-mediated recombination in the lung and in the myocardium of Ang II-treated CTR and *cmf*KO mice following Ang II exposure confirms numerous early findings. In our hands, cardiac *Postn* expression was restricted to fibrotic areas [50,81], while the expression of cGKI was efficiently ablated from collagen-rich myocardium in *Postn*iCre-driven *cmf*KO hearts (Figure 1D). In the absence of Ang II, however, and irrespective of the genotype of the mice, periostin was not detectable in the myocardium (Supplementary Figure S5G). Together, these findings allowed us to conclude that the primary source for cGKI expression in fibrotic lesions of the myocardium is attributable to *Postn^+^* cells. Consistent with this post-Ang II expression pattern of myocardial cGKI, cGKI protein levels were significantly lower in mixed *Postn^+^* CF/CMF cultures (Supplementary Figure S6B and C) obtained from *cmf*KO *versus* CTR hearts after the pathophysiological Ang II stimuli. This finding is consistent with other reports suggesting a functionally relevant role of the cGMP/cGKI axis in both CFs and CMFs [54,63].

Under physiological conditions, i.e., in the absence of Ang II, our in-depth analyses of CTR and *cmf*KO mice revealed no differences in survival, cardiac morphology, or heart function following TAM-induced *Postn*iCre-recombination (Figures 2 and 4H–K, Supplementary Figure S4A-F, S5E-G, S7 and S8). Conversely, in response to sustained Ang II stimulation, *Postn*iCre activation resulted in worse survival rates, CM hypertrophy, and an exaggeration of myocardial fibrosis in *cmf*KO hearts (Figures 1E and F and 2, S5A-F). Mice lacking cGKI globally develop hypertension likely due to a disruption of the NO-cGMP-cGKI cascade in VSMCs [85], while the contribution of CFs/CMFs to BP regulation is less clear. We, therefore, measured BP in freely moving *cmf*KO and CTR mice receiving TAM and Ang II according to the protocols described herein. Mean BP elevations by Ang II were significant as compared with the corresponding mice assessed prior to the Ang II infusion but yielded no gross differences between both genotypes (Figure 1F and G, S4K), suggesting that the higher susceptibility of CMF-specific cGKI KO hearts is due to local, i.e., intra-cardiac effects of the neurohormone Ang II, which promotes, besides its other adverse actions, the pro-fibrotic signaling in CF/CMF [86,87]. We were unable to clarify whether these cardiac effects of Ang II ultimately also explain the higher mortality in the +TAM+Ang II-treated *cmf*KO group (Figure 1E). However, we can rule out genotype-dependent effects on vascular remodeling processes in the aorta and in connection with renal fibrosis.

We were able to demonstrate a strong antiproliferative function of cGKI in CF as (i) the Ki-67 index in collagen-rich regions of the heart *in vivo* and (ii) the cell counts *in vitro* of mixed CFs/CMF cultures were amplified in the absence of the kinase. Thus, we conclude that the presence of cGKI is sufficient to counteract the expansion of *Postn^+^* cells under Ang II-stimulated conditions. This increase in cell proliferation rates explains the higher extent of myocardial fibrosis and an accumulation of type I and III collagens in the absence of CMF-specific cGKI *in vivo*. This is also confirmed by our *in vitro* analysis of the pro-fibrotic gene expression profile (Figure 4E), although the significance of these experiments is somewhat lower as all transcript quantifications were performed only after 24 h of the 8-Br-cGMP treatment in CF/CMF cultured in the absence of Ang II.

In our study, we used a moderate Ang II infusion protocol. Thus, it is not surprising that no gross signs of cardiac dysfunction, displayed trough unaltered %EF and %FS readings, were observed in the CTR mice. In contrast, both conventional M-mode and STE-based imaging uncovered a severe structure-related decline in global systolic function (%EF and %FS) and GLS, a measure of longitudinally oriented fibers highly prone to wall stress [88,89] in *cmf*KO mice. In both rat models and patients with HF, in particular, GLS was a reliable measure of LV function and a superior predictor of myocardial fibrosis and adverse cardiac outcomes [90-92]. Accordingly, the overall deformation capacity of the LV, as well as its regional deformation capacities and their rates, all of which are indices of myocardial stiffness and fibrosis, were markedly impaired in *cmf*KO hearts, which again fits their pro-fibrotic cardiac phenotype.

Larger CM cross-sectional areas, massive increase in fibrosis, and high CMF proliferation rates in *cmf*KO hearts did not correlate with larger HW or HW/TL ratios compared with CTR. Chronic exposure of Ang II has been shown to induce CM death [93-95], an effect that ultimately results in a loss of myocardial mass [96,97], which we confirmed by a higher count of apoptotic CMs in the absence of cGKI in *Postn^+^* cells. These conflicting actions of Ang II on cell growth, i.e., CM hypertrophy and CF/CMF proliferation *versus* cell death mechanisms apparently triggered a unique remodeling response within the *cmf*KO myocardium, ultimately resulting in an increase in muscle fibrosis, which could subsequently interfere with CM function and survival. Loss of structural integrity due to CM loss, in turn, also creates mechanical stress that may mediate CF-to-CMF activation. Thus, an urgent question that arises concerns the possible molecular interactions, paracrine and/or other mediators that are produced or released in a cGKI-dependent manner by *Postn* ^+^ CMF to be exchanged with the CMs to influence the function and ‘fate’ of the latter cell type. Two recent studies utilizing a FRET-based biosensor for cGMP live-cell imaging in CMs suggest that cGMP produced in response to NPs or upon stimulation of NO-GC in CFs can enter CMs via gap junctions [53,54]. Interestingly, this transfer has been observed for TCF21^+^ CFs, which are a prominent source for CMFs, i.e., *Postn* ^+^ CMF [13]. Although it is quite conceivable that the transfer of cGMP may contribute to the antifibrotic and antihypertrophic CF/CMF-to-CM cross-talk within the Ang II challenged heart, this hypothesis awaits experimental confirmation. While the extent of cGMP transfer under cardiac stress conditions may be stimulated by NO- and NP-dependent mechanisms, a contribution of cGKI seems to be rather unlikely because (i) the kinase acts exclusively downstream of cGMP production, and (ii) the intercellular transfer of cGKI itself from CF/CMF-to-CM via gap junctions seems neither very feasible nor likely. But how does the communication between myocardial CF/CMF and neighboring CMs works *in vivo* and how is cGKI involved in the transmission of the signals? Although this is obviously something that future studies must clarify in detail, it is well known that alterations in collagen levels, their disproportionate deposition within the myocardium, and even disbalances of collagen subtypes, promoted by CF/CMF, are directly associated with alterations in CM function. Besides these changes of the biomechanical environment (Figure 2A–C and S5A-D), the communication between both cell types might involve alterations in ionic/electrical coupling properties (transmitted through direct cell-to-cell, i.e., gap junctions) and biochemical factors that are secreted in a paracrine manner from CF/CMF [98,99]. Regarding the latter, our transcript analyses suggest that TGFβ1 and IL-6, two factors that can elicit CF-to-CMF conversion, ECM protein synthesis and CM hypertrophy, do not respond to cGMP/cGKI activation in *Postn* ^+^ CMF. Future studies should therefore consider other paracrine mediators such as fibroblast growth factor-2, ANP or BNP and their modulation upon pharmacological cGMP/cGKI stimulation as well as an in-depth analysis of the possible functions of this pathway in CF/CMF on Ca^2+^ handling mechanisms that ultimately may also affect neighboring CMs.

**Figure 5:**
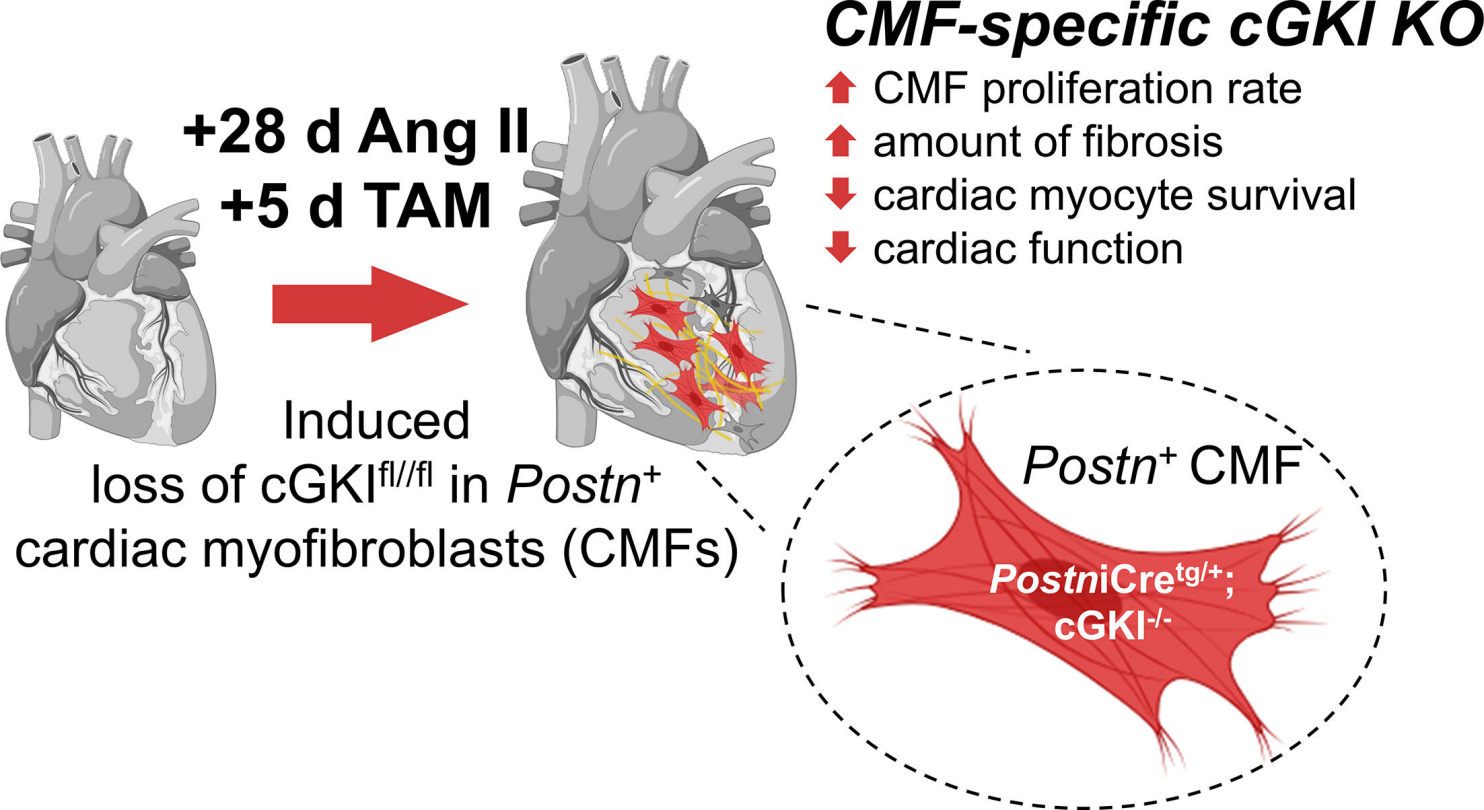
Summarized scheme of the results. As indicated, the loss of endogenous cGKI function in the cardiac myofibroblasts of male mice with angiotensin II exposure resulted in numerous deleterious effects and dysfunctions at the cellular and organ level (Created with BioRender.com).

In summary, the presented results highlight that the antifibrotic actions previously attributed to intrinsic NO/cGMP and NP/cGMP signaling pathways may require cGKI, specifically in *Postn^+^* CMFs . Interestingly, all *in vivo* effects reported herein were seen already in the absence of pharmacologically enhanced cGMP. At least for the Ang II-induced hypertensive heart, we, therefore, conclude that the endogenous response of counterregulatory mechanisms, which in the short term are usually compensatory and maintain cardiac function and perfusion of vital organs, is able to prevent deleterious CMF behaviors through cGKI activation (Figure 5). Nevertheless, it will be very interesting to examine whether anti-fibrotic effects of cGMP-elevating compounds such as vericiguat, BNP, and CNP also act via CMF-specific cGKI to enable positive changes in cardiac structure and/or function. Such approaches will also show how and whether the increase in catalytic activity of cGKI translates to kinase-regulated mechanism(s) downstream that directly counteract the stress response triggered by Ang II in *Postn* ^+^ CMFs.

## Summary

We herein show that the loss of cGKI in the *Postn* ^+^ subpopulation of CMFs *in vivo* leads to an exacerbation of the cardiac response to Ang II. These massive functional and structural changes were already observed after the genetic knockout of the kinase. From a clinical perspective, it will be interesting to investigate if an activation of the kinase in *Postn* ^+^ CMFs contributes to the clinical benefit of cGMP-elevating drugs such as vericiguat and neprilysin, which are prescribed to HFrEF patients. As exciting as this hypothesis is, it is far from being experimentally proven and, importantly, we have not yet exposed the Ang II-challenged *cmf*KO model to a respective treatment regime, i.e., to cGMP-elevating drugs. Also, it is presently not clear to what extent our findings regarding the antifibrotic roles of cGKI in *Postn^+^* CMF apply to post-MI hearts or to HF with preserved EF (HFpEF), which is a heterogeneous syndrome and proved to be a difficult entity to treat with many well-established HFrEF medications [100,101]. Yet, fibrosis is a major contributor to the pathogenesis of the ischemic myocardium and to HFpEF. We, therefore, anticipate that a better understanding of the cGMP/cGKI axis in *Postn^+^* CMFs will lead to important insight into the interaction between CMF and CM, which may ultimately foster our efforts to positively shape the cardiac microenvironment in the presence of various stress cues.

**Clinical Perspectives**

- Background
  Signaling by cGMP and cGMP-dependent protein kinase type I (cGKI) was previously shown to suppress cardiac fibrotic and dysfunction induced by angiotensin II. Whether this cascade counteracted the detrimental remodeling processes through a function that originates directly from cardiac myo-/fibroblasts remained unclear.

- Brief summary of the results
  Compared with their respective control littermates, tamoxifen-induced cardiac myofibroblasts-specific cGKI knockout (*cmf*KO) mice developed significantly increased cardiomyocyte cross-sectional areas, a marked increase in myocardial fibrosis, as well as a significant structure-related distortion of global systolic heart function.

- Potential significance of the results to human health and disease
  Pharmacological modulation of cardiac myofibroblast-specific cGKI should provide new opportunities to optimize existing treatments aimed at improving or even reversing hypertensive heart disease, one of the major causes of heart failure.

## Supplementary material

Online supplementary material

## Data Availability

Requests for resources and reagents can be addressed directly to the Lead contact, Robert Lukowski (robert.lukowski@uni-tuebingen.de). All data generated or analyzed during this study that are not included in this published article and its supplementary information are available from the corresponding author on reasonable request.

## Abbreviations

Ang II: angiotensin II
CF: cardiac fibroblast
CM: cardiomyocyte
CMF: cardiac myofibroblast
CTR: control
CVDs: cardiovascular diseases
HF: heart failure
*Postn*: periostin
TAM: tamoxifen
VSMCs: vascular smooth muscle cells
cGMP: 3′,5′-cyclic guanosine monophosphate
*cmf*KO: CMF-specific cGKI knockout
