# Supplementary material for: Angiotensin II-induced cardiac fibrosis and dysfunction are exacerbated by deletion of cGKI in periostin + myofibroblasts
Source: Clin Sci (Lond). 2025 May 28;139(11):507–26. doi: 10.1042/CS20241204 (PMC12247847; doi:10.1042/CS20241204)
Supplement: Online supplementary material [file cs-139-11-CS20241204-s001.doc]

**Angiotensin II-induced cardiac fibrosis and dysfunction are attenuated by cGKI signaling in periostin^+^ myofibroblasts**

Melanie Cruz Santos^1^, Lena Birkenfeld^1^, Thomas Pham^1^, Selina Maier^1^, Katharina Paulus^1^, Lena Ullemeyer^1^, Amelie Knauer^1^, Clement Kabagema-Bilan^1^, Natalie Längst^1^, Anna Roslan^1^, Nina Wettschureck^2^, Meinrad Gawaz^3^, Fumito Ichinose^4,5^, Robert Lukowski^1,§^

^1^University of Tübingen, Department of Pharmacology, Toxicology and Clinical Pharmacy, Institute of Pharmacy, Tübingen, Germany

^2^Max-Planck-Institute for Heart and Lung Research, Department of Pharmacology, Bad Nauheim, Germany

^3^University of Tübingen, Department of Cardiology and Angiology, University Hospital Tübingen, Tübingen, Germany

^4^Harvard Medical School, Boston, United States of America

^5^Massachusetts General Hospital, Department of Anesthesia, Critical Care and Pain Medicine, Boston, United States of America

^§^Corresponding author:

Robert Lukowski

Experimental Pharmacology

Department of Pharmacology, Toxicology and Clinical Pharmacy

Institute of Pharmacy

University of Tübingen

Tel. +49 7071 29 74550

Fax. +49 7071 29 2476

E-Mail: robert.lukowski@uni-tuebingen.de

Supplemental information: Figures and Figure Legends (SFig. 1-8)
 Methods

Reagents and Tools

References

Values and statistics (Fig. 1-4)

**Supplemental Figures and Figure Legends**

##
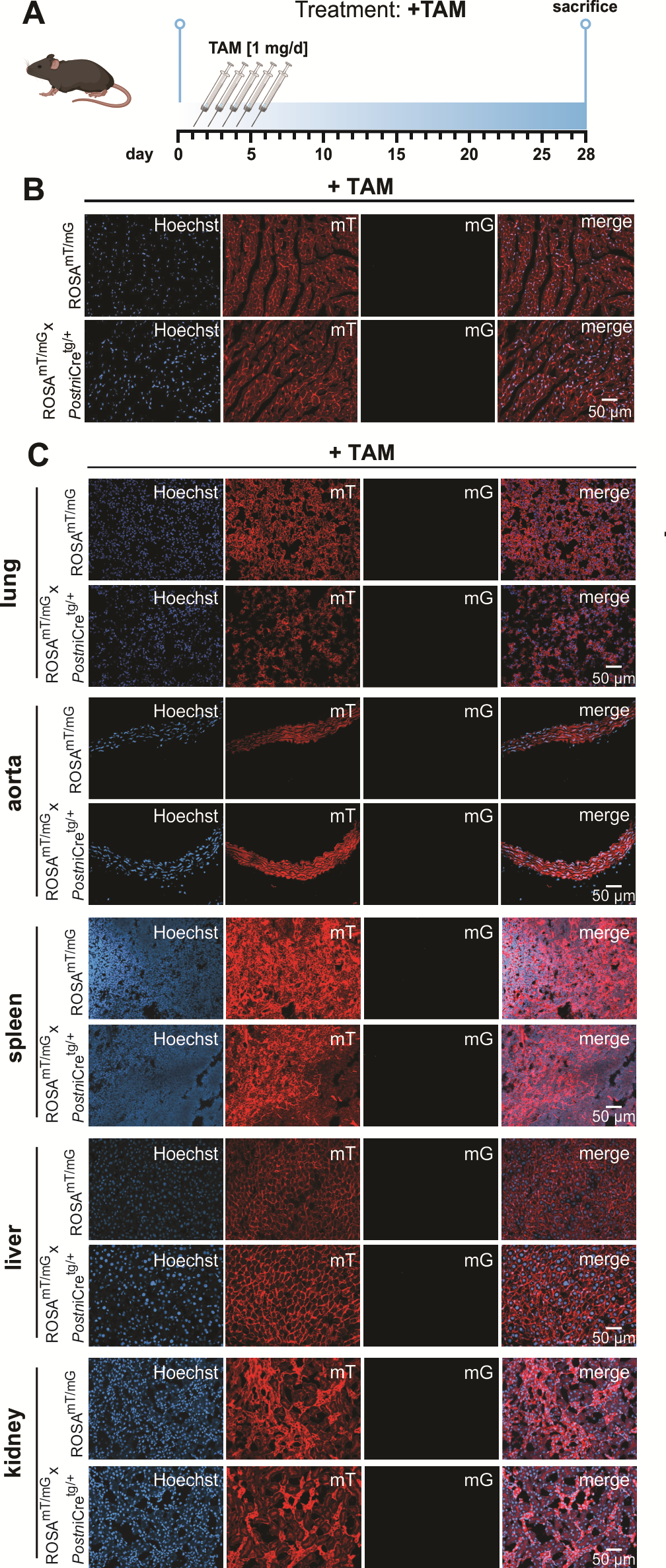


## SFigure 1: *Analysis of a two-color fluorescent Cre-reporter mT/mG for in vivo monitoring of TAM-induced CMF-specific PostniCre recombination*

(**A**) Experimental setup in chronological order performed for the induction of the TAM-induced *Postn*iCre-mediated recombination under baseline i.e., physiological conditions. Created with [BioRender.com](https://biorender.com/). (**B**) The two-color fluorescent ROSA^mT/mG^ Cre reporter strain was intercrossed with transgenic *Postn*iCre^tg/+^ mice, and cell-specific *Postn*iCre-mediated recombination was evaluated in experimental mice subjected to the protocol shown in (A). Expression of the membrane-targeted green fluorescent (mG) protein, which would indicate Cre-mediated recombination, was not detectable in cardiac cryosections obtained from TAM-treated double transgenic ROSA^mT/mG^ x *Postn*i*Cre*^tg/+^ mice. Heart slices from ROSA^mT/mG^ Cre reporter mice served as control. n = 3 heart slices of N = 3 mice per genotype were evaluated. (**C**) Representative fluorescent images of selected organs obtained from ROSA^mT/mG^ Cre reporter as well as double transgenic ROSA^mT/mG^ x *Postn*iCre^tg/+^ mice. Under physiological conditions (+TAM, SFig. 1A), ubiquitous mT-protein expression, indicating absence of green-fluorescent mG protein, was detectable in both genotypes. Other organs obtained from double-transgenic as well as from ROSA^mT/mG^ Cre reporter lacking Cre-recombinase continued to express the red fluorescent mT protein. Images shown are representative for n = 3 tissue slices per organ obtained from N = 3 mice per genotype per condition.

##
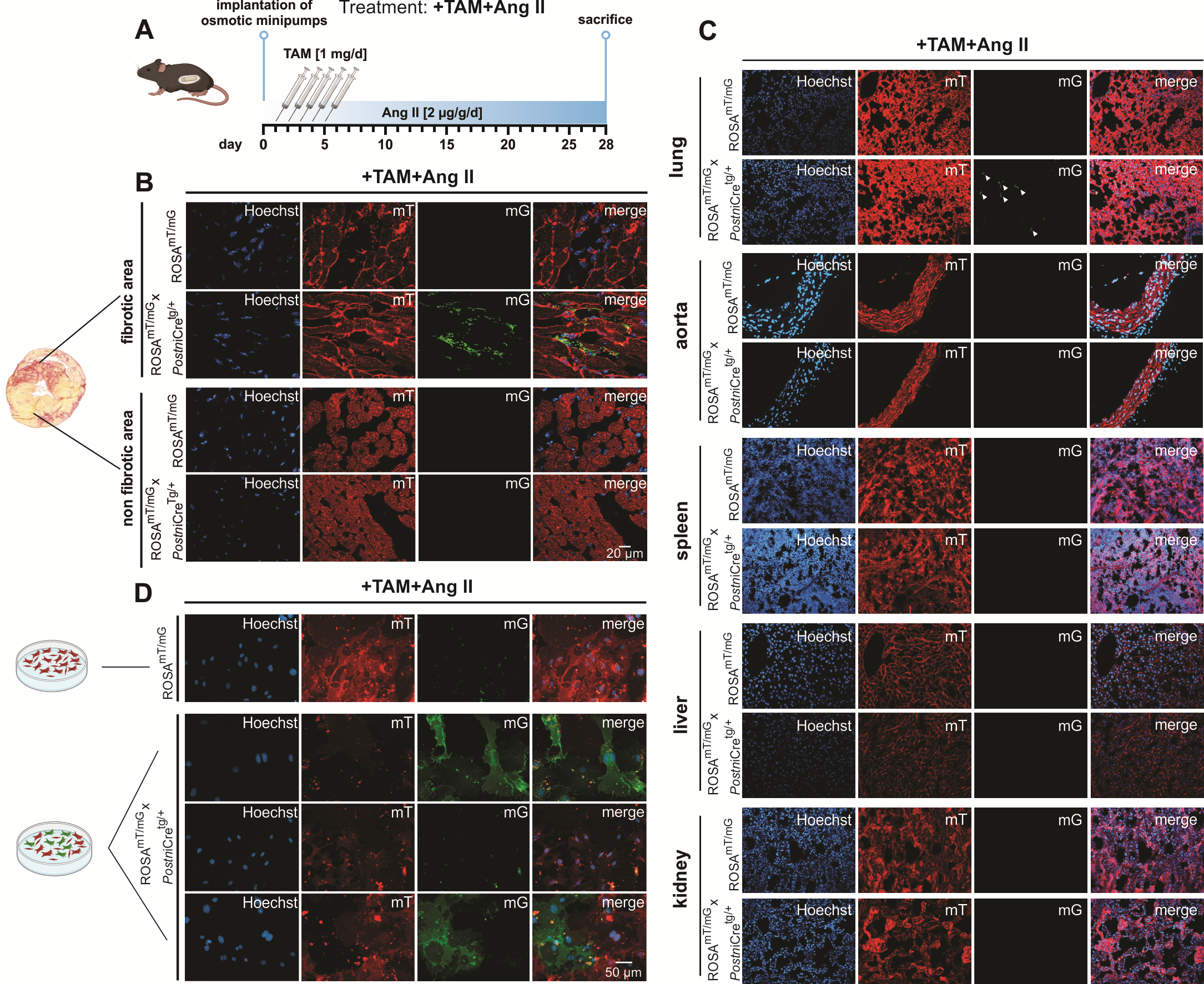


## SFigure 2: *Comprehensive characterization of the tissue-specific PostniCre-mediated recombination in the presence of Ang II*

(**A**) Experimental setup for the TAM-induced *Postn*i*Cre*-recombination followed by prolonged Ang II exposure to provoke cardiac remodeling. Created with [BioRender.com](https://biorender.com/). (**B**) Following this protocol, hearts harvested from ROSA^mT/mG^ reporter mice and double transgenic ROSA^mT/mG^ x *Postn*i*Cre*^tg/+^ mice were stained with PSR to identify fibrotic regions. In subsequent sections, Cre-mediated recombination was observed exclusively in cells located in the interstitial spaces of cardiomyocytes, as indicated by the switch from membrane-targeted red fluorescent protein (mT) to expression of mG. Cardiomyocytes continued to express the red fluorescent mT-protein. Heart slices obtained from transgenic ROSA^mT/mG^ mice revealed ubiquitous expression of the red fluorescent mT-protein and served as controls. n = 3 heart slices of N = 3 per genotype. (**C**) Representative fluorescent images of selected organs obtained from ROSA^mT/mG^ Cre reporter as well as double transgenic ROSA^mT/mG^ x *Postn*iCre^tg/+^ mice. Following prolonged hypertrophic stimulation with Ang II (+TAM+Ang II, SFig. 2A), expression of the green-fluorescent mG protein was seen exclusively and only in a minority of cells in the lungs isolated from double transgenic mice (white arrows). Other organs obtained from double-transgenic as well as from ROSA^mT/mG^ Cre reporter lacking Cre-recombinase continued to express the red fluorescent mT protein. Images shown are representative for n = 3 tissue slices per organ obtained from N = 3 mice per genotype per condition. (**D**) Primary CFs/CMFs obtained from transgenic ROSA^mT/mG^ mice lacking the *PostniCre* transgene*,* exclusively express the red fluorescent mT-protein following +TAM and +Ang II treatment as outlined in (A). Cre-mediated excision of the loxP-flanked red fluorescent mT protein leading to the expression of mG protein, was only detectable in CMFs isolated from ROSA^mT/mG^ x *Postn*i*Cre*^tg/+^ upon *in vivo* challenge with +TAM and +Ang II for 28 d according to the protocol shown in (A). Representative images obtained from one ROSA^mT/mG^ and one ROSA^mT/mG^ x *Postn*i*Cre*^tg/+^ heart are shown. N = 3 primary CF/CMF cultures per genotype were analyzed. Cell nuclei were visualized with Hoechst.

##
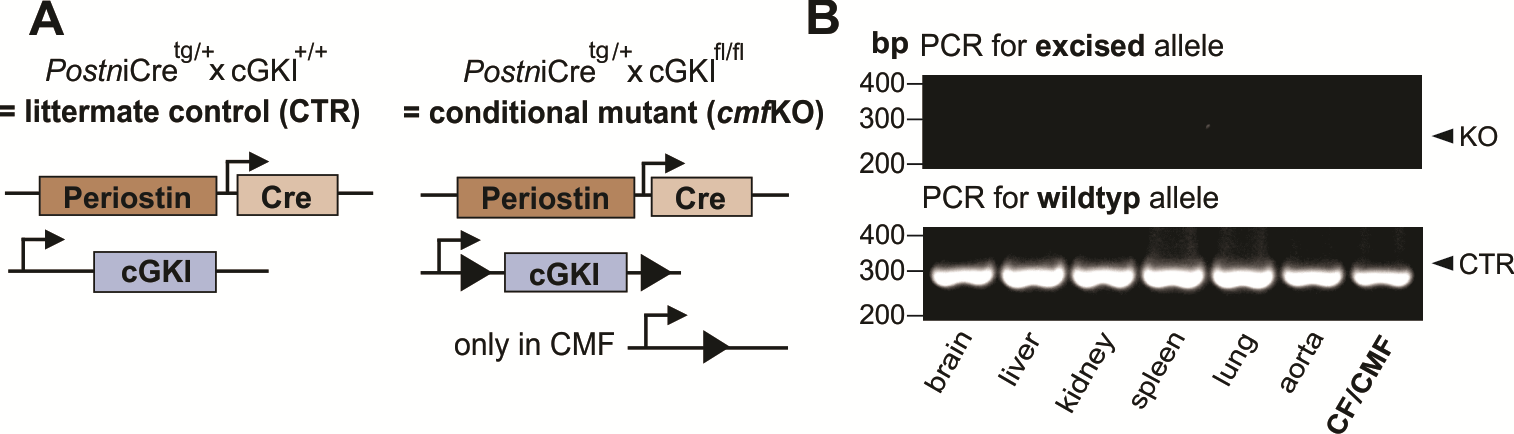


## SFigure 3: *Generation of the CMF-specific cGKI KO (cmfKO) and corresponding CTR littermates*

(**A**) The *Postn*iCre^tg/+^ mouse strain expressing a TAM-inducible CreER^T2^ (Cre) recombinase under the control of the *Postn* promotor was used to generate a CMF-specific cGKI KO. Expression of *Postn* has previously been reported to correlated with the activated state of CFs following pathophysiological stimuli (1). The progeny resulting from mating *Postn*iCre^tg/+^ x cGKI^fl/+^ with heterozygous cGKI^fl/+^ mice led to the generation of experimental mice with the desired genotypes i.e., *Postn*iCre^tg/+^ x cGKI^+/+^ (CTR) and *PostniCre*^tg/+^ x cGKI^fl/fl^ (*cmf*KO) littermates specifically lacking cGKI in CMFs. (**B**) Genomic PCR analysis was conducted with DNA samples extracted from indicated tissues as well as primary CFs/CMFs of Ang II-treated CTR mice. As expected, exclusively the wildtype ([+]; 284 bp) allele was detected in animals treated with TAM for five consecutive days.

##
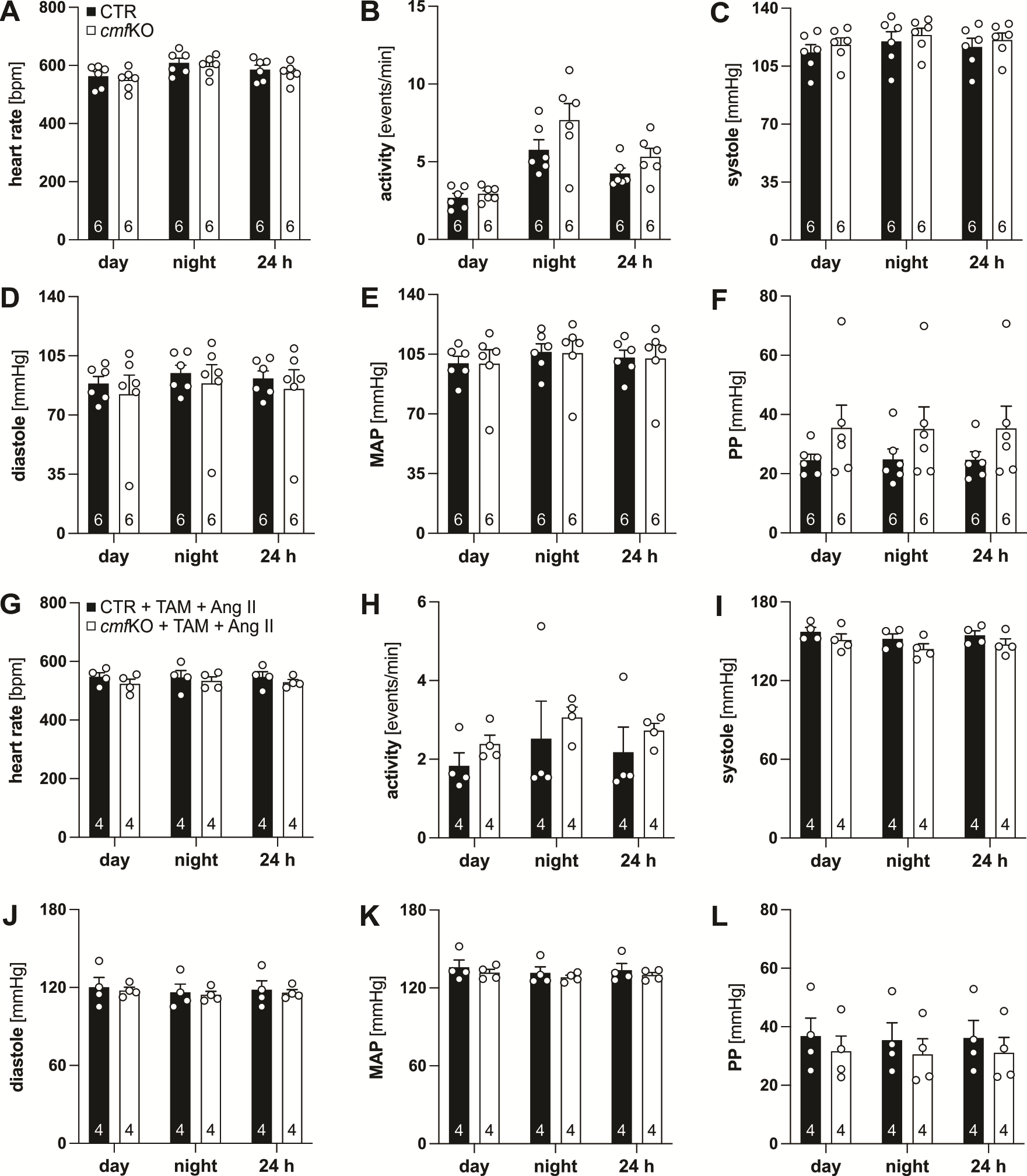


## SFigure 4: *Telemetric blood pressure measurements in CTR and cmfKO mice under basal (A-F) and +TAM +Ang II infusion (G-L) conditions*

Telemetric recordings exhibited no genotype-related alterations in (**A**) heart rate, (**B**) locomotor activity, (**C**) systolic and (**D**) diastolic blood pressure (BP). Accordingly, (**E**) mean arterial pressure (MAP) and (**F**) pulse pressure were similar in CTR und *cmf*KO mice prior to TAM-induced Cre-recombination and Ang II infusion. The distinct parameters were continuously recorded from the same experimental animal over 72 h with a 12-hour light-dark cycle. Plotted are the daytime, nighttime, and the average 24-h values of N = 6 mice per genotype. Upon implantation of the osmotic minipumps and 5 days of TAM injections (Suppl. Fig. 2A), BP measurements were recorded continuously for the following seven days in N = 4 mice per genotype. Throughout this period, TAM-mediated Cre recombination did not cause genotype-related differences in **(G)** heart rate, **(H)** locomotor activity patterns**, (I)** systolic, and **(J)** diastolic PB**.** Accordingly, **(K)** MAP and **(L)** pulse pressure were not significantly different between genotypes at any time of day or night. **Statistics**: Multiple unpaired t-test corrected for multiple comparison using the Holm-Šídák method **(A**, **C-E, G**, **I-L)**, multiple Mann-Whitney test corrected for multiple comparison using the Holm-Šídák method **(B**, **F**, **H)**.

##
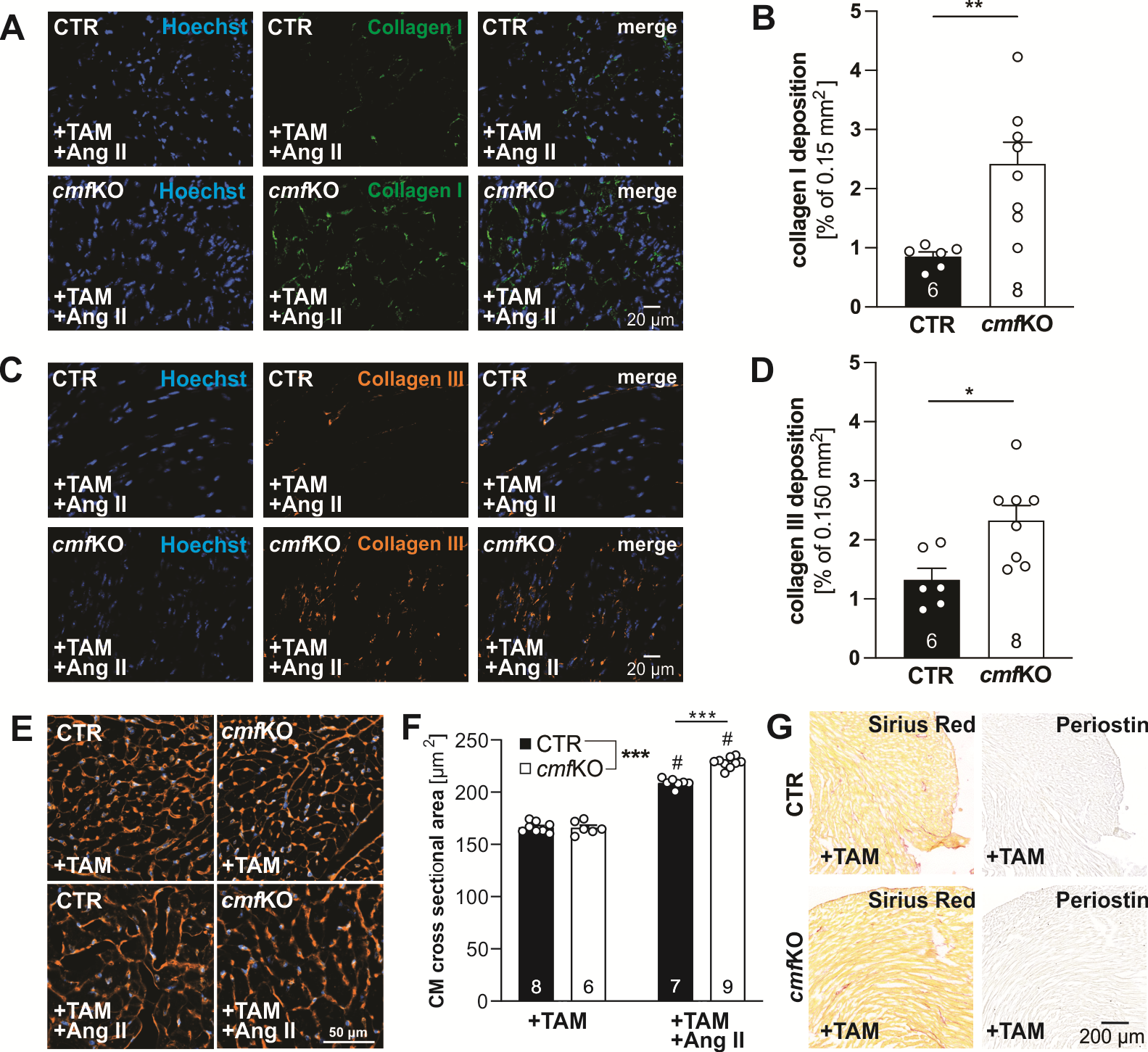


## SFigure 5: *Quantification of collagen deposition and CM cross section areas in ±Ang II and +TAM treated CTR and cmfKO hearts*

(**A-D**) Representative (**A**) type I and (**C**) type III collagen immunofluorescence of +TAM and +Ang II treated CTR (for both staining’s N = 6) and *cmf*KO hearts (for both staining’s N = 8). Quantification of (**B**) collagen I and (**D**) collagen III deposition expressed as percentage of the observed myocardium revealed a significant increase in *cmf*KO compared to CTR hearts. Evaluation of n = 4 consecutive heart slices of three distinct section (IV-VI) per heart (N = 6-8 per genotype for type I and III collagen, respectively). **Statistics:** (B, D) Unpaired student t-test with **P*<0.05 and ***P*<0.01. (**E**) Representative fluorescently labeled wheat germ agglutinin (WGA)-staining of cardiac cryosections obtained from CTR and *cmf*KO after either +TAM or +TAM+Ang II treatment. (**F**) CM cross-sectional areas significantly increased in both genotypes after the Ang II infusion (CTR, N = 7; cmfKO, N = 9) compared to the respective control groups that received +TAM only (CTR, N = 8; cmfKO, N = 6). The amount of hypertrophy induced by Ang II resulted in lager cross section areas in the absence of cGKI expression in *cmf*KO hearts. **Statistics:** Two-way ANOVA followed by Tukey's multiple comparison test with # indicating significant difference (*P*<0.001) between CTR+TAM *versus* CTR+TAM+Ang II and *cmf*KO+TAM *versus cmf*KO+TAM+Ang II groups and ****P*<0.001 for the difference between the genotypes as indicated. (**G**) Sirus red and antibody-based anti-periostin stain of representative myocardial cryosections obtained from CTR and *cmf*KO mice treated with +TAM only. Neither fibrotic lesions nor periostin expression were detectable under these experimental conditions, i.e. in the absence of Ang II infusion. One of three representative heart slices from n=3 mice are shown.


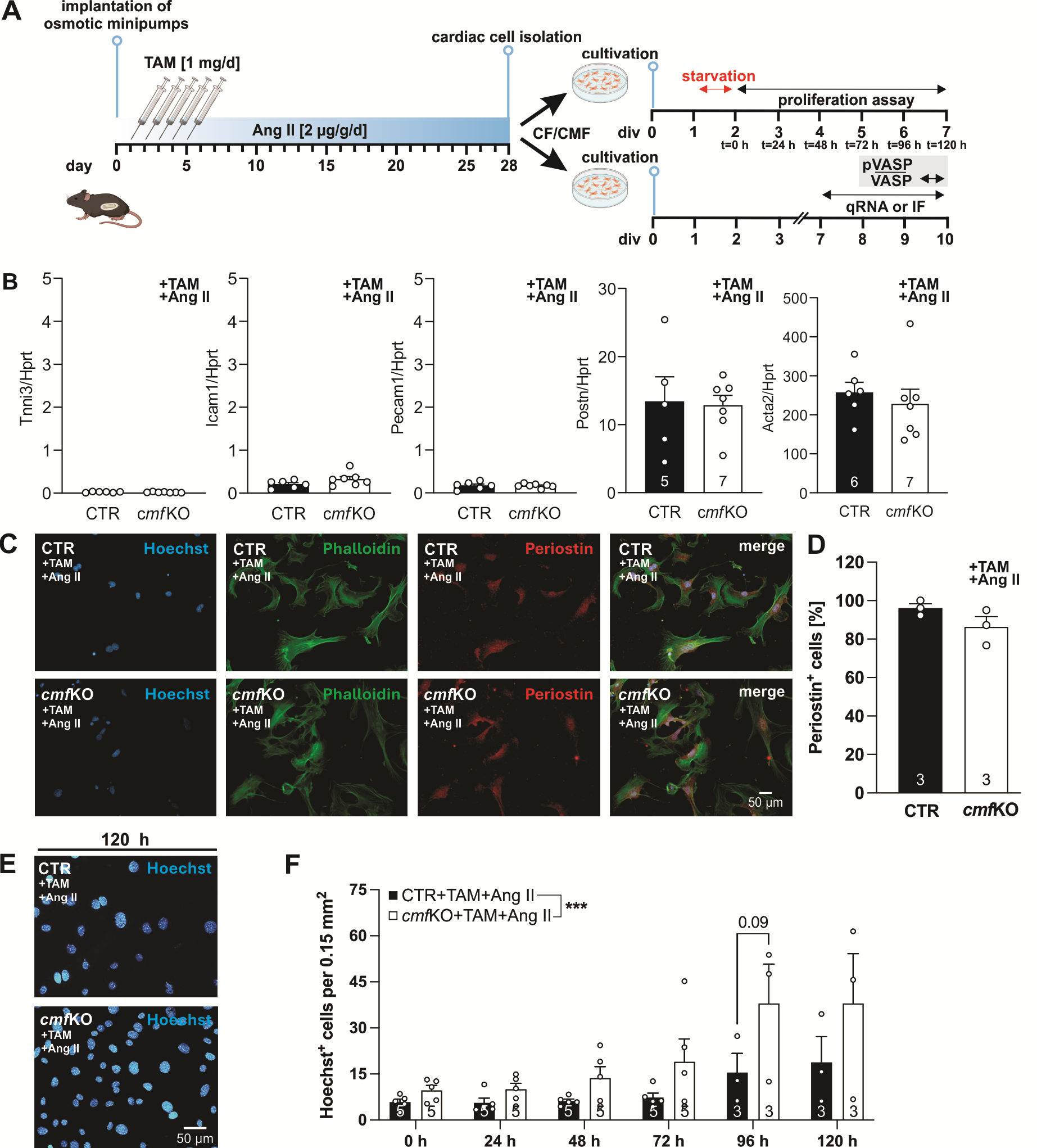


## SFigure 6: *Evaluation of cell purity and proliferative properties of* *CF/CMF primary cell cultures derived from TAM and Ang II-treated CTR and cmfKO hearts*

(**A**) Schematic representation of the experimental protocol. CFs/CMFs were isolated from +TAM and +Ang II-treated CTR and *cmf*KO mice (left) to establish primary cell cultures (right). Time sequence for cell cultures used to perform (**B**) qRT-PCR and (**C-D**) immunofluorescence (IF)-based analyses or (**E-F**) the proliferation assay. Created with [BioRender.com](https://biorender.com/). (**B**) Transcript analysis of CFs/CMFs (at d7 to d10) in culture following the protocol shown in (A, lower panel) revealed low expression levels for marker genes preferentially expressed by CMs (Tnni3/cTNI) and endothelial cells (Icam1/ICAM-1). Also, Pecam1/PECAM-1, a broad cell surface marker of hematopoietic and immune cells including platelets, was hardly detectable, Postn/Periostin and αSM-actin (Acta2), key markers of cardiac myofibroblasts, were expressed at much higher levels. HPRT expression was used as reference and to normalize the data using the 2^−△CT^ method (n=5-7 primary CF/CMF cultures / hearts per genotype) (**C**) IF analyses performed on the same cultures assessed in (B) revealed that (**D**) >85% of the CTR and cmfKO cells were periostin positive indicating CMF features (n=3 primary CF/CMF cultures / hearts per genotype). **Statistics:** Means +SEM were compared by an unpaired t test revealing no differences between genotypes. (**E**) For the proliferation assay (A, upper panel) CFs/CMFs were allowed to adhere to the bottom of the culture surface for 24 h, followed by a starvation period of 24 h in FBS free medium to enrich cells in the G_0_/G_1_ phase of the cell cycle. Following this protocol representative images of a nuclear staining of primary CFs/CMFs at t = 120 h were acquired. (**F**) Quantification of the number of Hoechst^+^ cells per 0.15 mm^2^ area detected at the defined time points (t = 0 - 120 h) as indicated. Throughout the observation period, a significantly higher CF/CMF count was registered in primary cell cultures obtained from *cmf*KO compared with CTR mice. **Statistics**: Two-way ANOVA followed by Šídák's multiple comparisons test with significant difference ****P*<0.001 between genotypes.

##
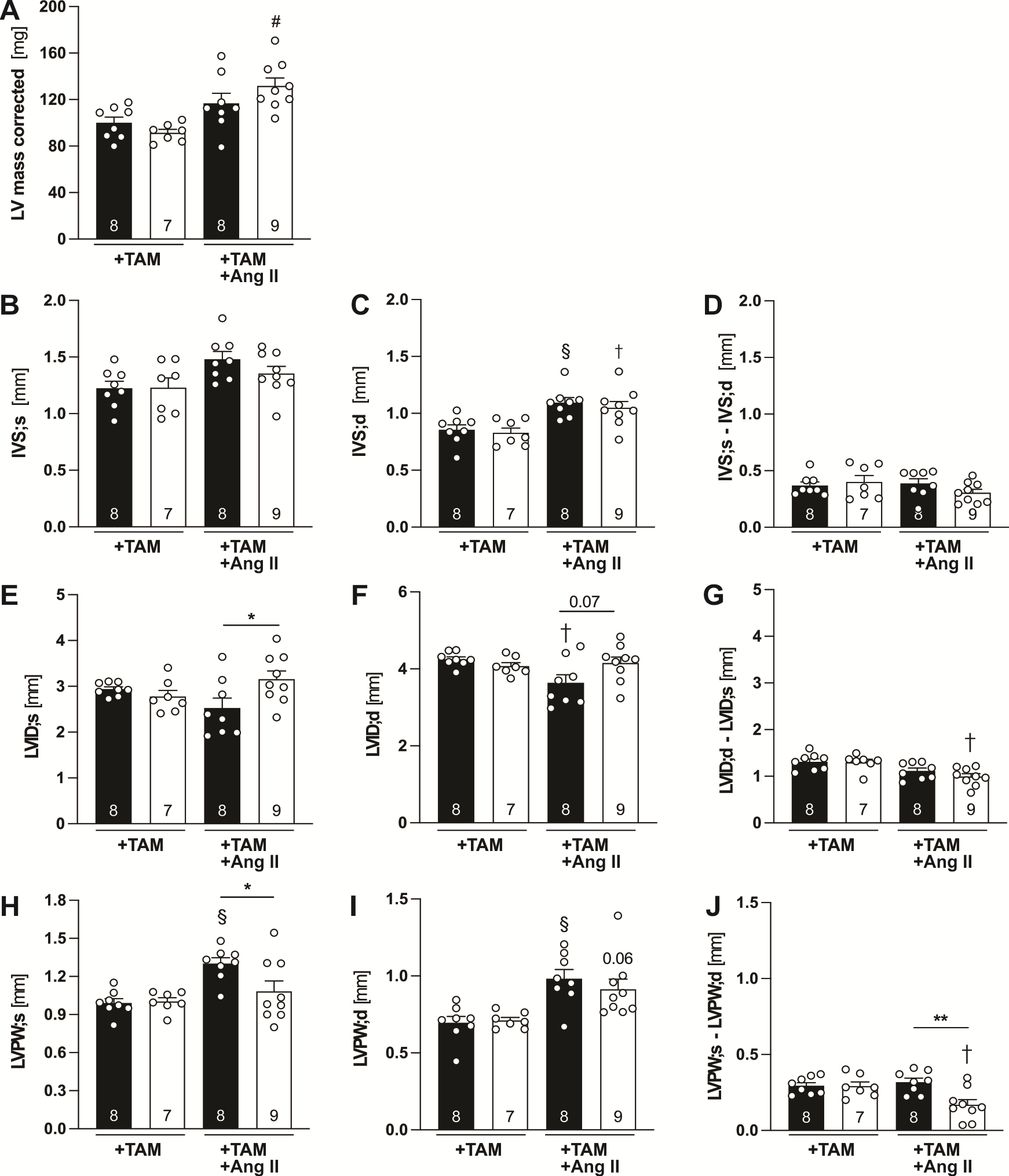


## SFigure 7: *LV morphology of cmfKO and CTR mice 28 days after Ang II treatment*

Data were acquired using conventional M-mode echocardiography in the PSLAX view to evaluate morphological parameters of the LV under physiological conditions (CTR: N = 8 and *cmf*KO: N = 7) and upon chronic Ang II exposure in TAM-induced CTR (N = 8) and *cmf*KO (N = 9) mice. (**A**) While TAM and Ang II-treated CTR mice exhibited only a trend towards an increase in LV mass_corrected_ compared to the corresponding TAM group, this parameter was significantly higher in TAM and Ang II challenged compared to unchallenged *cmf*KO mice. While (**B**) end-systolic IVS dimension remained largely unaltered in both genotypes, (**C**) the end-diastolic thickness of the IVS was significantly increased in CTR and *cmf*KO groups in response to prolonged Ang II infusion compared to the respective TAM controls. (**D**) However, the difference between maximal thickening of the IVS in systole and maximal relaxation in diastole was not significant between the four analyzed groups. (**E**) End-systolic LVID increased significantly in the *cmf*KO hearts compared to the CTR hearts after Ang II treatment, although end-systolic LVID values of both groups were similar to TAM-treated controls of the same genotype. (**F**) Compared to CTR mice exclusively treated with TAM, end-diastolic LVID was significantly diminished in the Ang-II treated CTR , but not different to Ang-II treated *cmf*KO mice. (**G**) The difference between the largest LVID measured at the end of diastole and the smallest LVID measured at the end of systole was diminished in both genotypes compared with the corresponding TAM-treated control groups, but only the comparison among the *cmf*KO groups reached the statistical significance level. (**H**) Ang II-treated CTR mice exhibited a significant increase in end systolic LVPW thickness compared to both their respective control group and the Ang II-treated *cmf*KO and, while (**I**) both genotypes developed a strong increase in end diastolic LVPW thickness upon Ang II treatment. (**J**) These end diastolic and systolic differences in LVPW ultimately resulted in a smaller range of LVPW thickness in systole and upon maximal relaxation in diastole in Ang II-treated *cmf*KO compared to the Ang II-treated CTR mice and the corresponding TAM-treated *cmf*KO group. **Statistics**: Two-way ANOVA followed by Tukey's multiple comparison test with significant difference **P*<0.05 and ***P*<0.01 between genotypes and †P<0.05; §P<0.01 and #P<0.001 revealing differences between the distinct treatments (+TAM *versus* +TAM+Ang II) within the same genotype. For panel (**G**), which was analyzed using the Kruskal-Wallis test followed by the Dunn test for multiple comparisons, a significant difference at †P<0.05 in the *cmf*KO mice between the +TAM and the +TAM+Ang II treatment was detected.


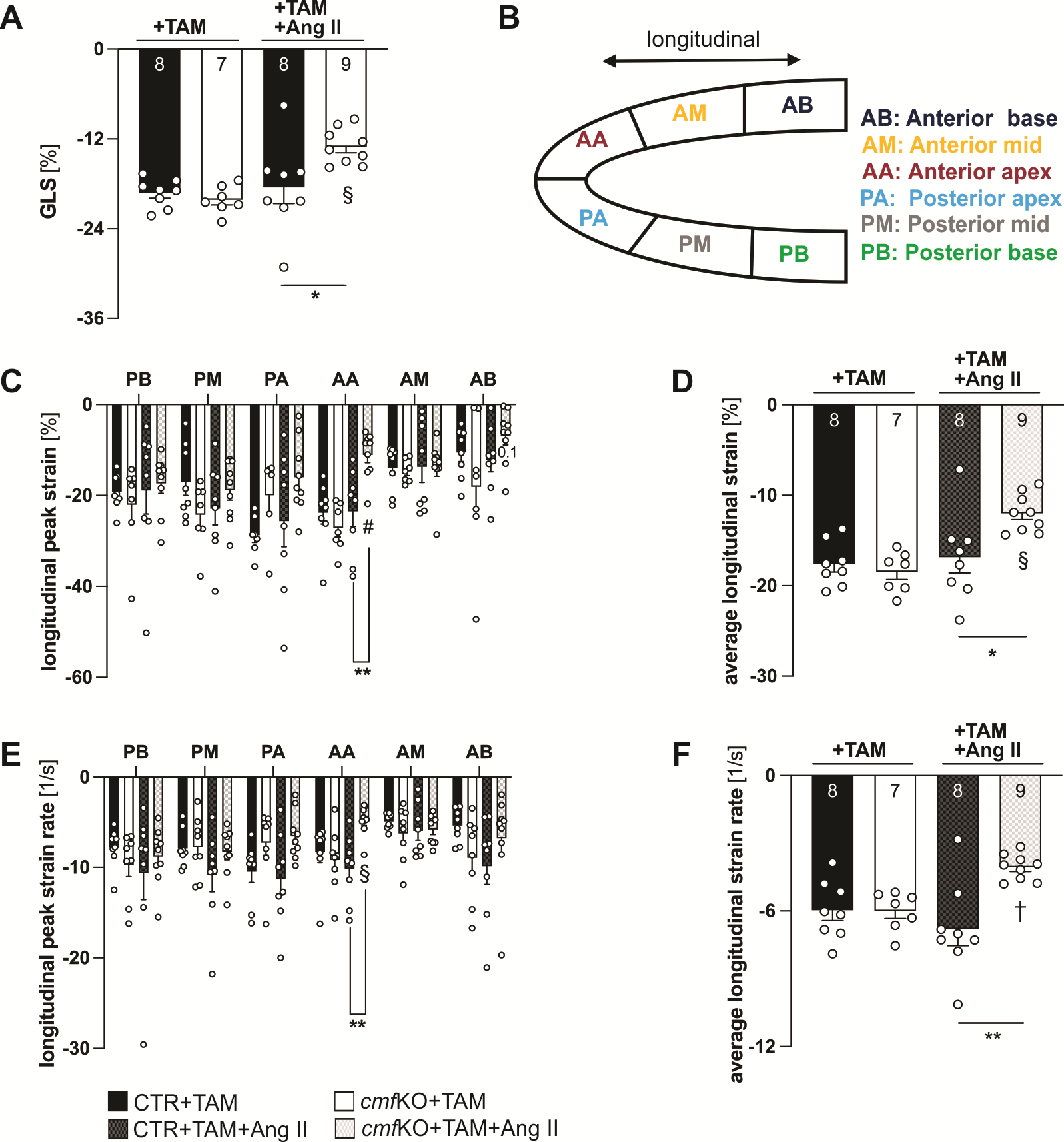


## SFigure 8: *cmfKO hearts displayed a significant deficiency in global and regional LV deformation capacity*

## Strain analysis by speckle tracking echocardiography (STE) acquired in the PSLAX B-Mode view were performed to quantify global and regional left ventricular motion abnormalities. (A) Using LV wall trace analysis for the calculation of the global longitudinal strain (GLS), significantly increased values were obtained from Ang II-treated *cmf*KO mice compared to both Ang II-treated CTR mice and unchallenged hearts from the corresponding *cmf*KO control group, which indicates a severe deterioration in cardiac contractility. (B) In order to examine quantitative indices of the regional deformation capacity, the hearts were automatically divided into six different regions by the VevoStrain software. Schematic representation modified from Frankenreiter et al. (2017) (2). (C) Summary data of region-specific longitudinal peak strain and (D) the average longitudinal strain as measures of the deformation of the LV wall from a relaxed to a contracted condition. Ang II-treated *cmf*KO mice exhibited a significantly impaired LV deformation capacity compared with Ang II-treated CTR mice and the corresponding TAM-treated control group. (E) Summary data of region-specific longitudinal peak strain rate and (F) average strain rate of the LV wall. (F) In line with previous results, Ang II treatment caused a significant increase of the longitudinal change in deformation with respect to time in *cmf*KO hearts compared to the CTR mice as well as to the TAM-treated corresponding control group. Statistics: Two-way ANOVA followed by Tukey’s multiple comparisons test (A, C [PB, PM, AA, AB], D, E [PM, AM, AB], F), Kruskal-Wallis test followed by the Dunn test for multiple comparisons (C [PA, AM], E [PB, PA, AA]) with **P*<0.05 and ***P*<0.01 indicating significant difference between genotypes and †P<0.05 and §P<0.01 revealing differences between distinct treatments (+TAM *versus* +TAM+Ang II) within the same genotype.

**Supplemental Methods**

## Survival curves

The Kaplan-Meier survival curve was generated based on the survival rate of CTR and *cmf*KO mice upon TAM i.p. and in the TAM i.p. group receiving Ang II for 28 days. Although animal welfare was accurately assessed and regularly reviewed on the basis of pre-established criteria and early end point definition in all groups and during all procedures, spontaneous deaths occurred in both genotypes as a result of the combined +TAM and +Ang II treatment, without any detectable changes in appearance, behavior or symptoms upfront. Only animals that reached the endpoint (28 d) were also included in all subsequent analyses.

## Preparation of cryosections

## After the mice were euthanized with CO_2_, the hearts were harvested and retrogradely perfused via the aorta with ice-cooled Dulbecco's Balanced Salt Solution (DPBS, Thermo Scientific #14190144) using a Langendorff perfusion system to remove blood clots as well as further cellular debris (3). The resulting purified hearts were fixed in 4% paraformaldehyde (PFA) in DPBS at 4°C for 4 h before being washed thrice with DPBS. Following an ascending sucrose gradient (5% for 1 h, 10% for 12 h, 20% for 24 h - solved in DPBS), hearts were placed in Neg-50^TM^ (Epredia, #6502) embedding medium and stored at -80°C. Hearts were kept at -20°C for 30 min before 10 µm transverse cryosections were generated using a Microm HM 560 microtome (Thermo Scientific) and collected on glass slides (Epredia Superfrost^TM^ Plus Adhesion Microscope Slides #J1800AMNZ). To detect tissue-specific Cre recombination, additional organs (aorta, lung, spleen, kidney, liver) were excised from ROSA^mT/mG^ and ROSA^mT/mG^ x *PostniCre*^Tg/+^ mice and immediately fixed in 4% PFA, applying the same protocol as described above. For cardiac outcome analyses, hearts were divided into 8 equidistant regions, starting from the apex to the base of the hearts, with 20 consecutive heart slices collected from each region.

## Immunofluorescence

In order to detect cell- and tissue-specific Cre-mediated recombination, thawed tissue cryosections obtained from ROSA^mT/mG^ Cre reporter and double transgenic ROSA^mT/mG^ x *Postn*iCre^tg/+^ mice were initially permeabilized using 0.3% Triton-X-100 (Carl Roth #3051) in DPBS for 30 min, followed by three washing steps with DPBS. Nuclei staining by 0.1% Hoechst 33432 (Thermo Scientific #62249) in PermaFluor aqueous mounting media (Thermo Scientific #TA-030-FM) allowed visualization of tissue sections using a fluorescent Apotome microscope (Zeiss).

To examine collagen deposition as well as to quantify Ki67 positive nuclei in Ang II remodeled hearts, thawed cardiac cryosections derived from CTR and *cmf*KO animals were exposed to 0.3% Triton-X-100 in DPBS for 30 min. By applying a chemical cocktail consisting of 3% H_2_O_2_ and 50 mM NaOH for 90 min, autofluorescence of cardiac cryosections was diminished (4). After several washing steps in DPBS, unspecific antibody binding was prevented using a blocking buffer (DPBS supplemented with 2% Glycerol, 5% NGS, 0.3% Triton-X-100, 2% BSA and 50 mM NH_4_Cl) for 1 h at RT. Heart sections were incubated in primary antibodies against either collagen I (1:1000, Abcam #ab6308) and Ki67 (1:1000, Cell Signaling Technology #9129) or collagen III (1:500, Abcam #184993) diluted in blocking buffer overnight at 4°C. Subsequently, sections were washed with 0.01% Triton-X-100 in DPBS and incubated with secondary antibodies (Goat anti-Mouse IgG1 Alexa Fluor 488 1:2000; Goat anti-Rabbit Alexa Fluor 555 1:1000; Goat anti-Rabbit Alex Fluor 647 1:1000) and Hoechst 33432 (1:1000) for 2 h at RT. After renewed multiple washing steps with DPBS, sections were mounted in PermaFluor aqueous mounting media. ImageJ software (ImageJ 1.53) was employed to quantify Ki67-positive nuclei as well as collagen I and III deposition as a percentage of the respective heart area.

The *In Situ* Cell Death Detection Kit (Roche #12156792910) based on terminal deoxynucleotidyl transferase-mediated deoxyuridine triphosphate (dUTP) nick end labelling (TUNEL)-assay was employed to detect and quantify apoptotic CMs death (5). Prior to TUNEL-staining, immunofluorescence staining against troponin I (1:100, Cell Signaling Technology #4002S) was performed according to the previously described protocol to specifically identify CMs in heart slices obtained from TAM- and Ang II-treated CTR and *cmf*KO mice. After incubation with the secondary antibody (Goat anti-rabbit Alexa Fluor 488; 1:1000) and Hoechst 33432 (1:1000), the heart sections were washed thrice in DPBS before TUNEL reaction solution was applied for one hour at 37°C in a humidity chamber according to the manufacturer's instructions. Subsequently, the samples were washed again in DPBS, air-dried, and embedded in PermaFluor. Heart sections were imaged using a fluorescent Apotome microscope (Zeiss) and TUNEL-positive CMs quantified as percentage of the total amount of CM nuclei counted in the respective image section using ImageJ.

## Histochemical staining procedures

Hematoxylin and eosin (H&E) staining was conducted to visualize cardiac morphology as well as to determine CM cross-sectional areas in heart slices obtained from CTR or *cmf*KO animals under basal conditions or after Ang II treatment as previously described (6, 7). Thawed heart cryosections were initially rehydrated in descending concentrations of ethanol (100%, 90%, 70%, 50%) and washed thrice in ddH_2_O before being incubated for 7 s in Harris Haematoxylin solution (Carl Roth #X903.2). Upon washing in ddH_2_O to remove excess staining solution, cryosections were rinsed in 0.1% ammonia solution in order to intensify nuclear staining. The sections were washed again in tap water prior to the incubation in acidic Eosin-Y solution (ddH_2_O supplemented with 20% Eosin G-solution and one drop 100% acetic acid) for 8 min. Excess staining solution was once again removed by a further washing step in tap water. Following dehydration of the cryosections by increasing concentrations of ethanol (80%, 100%) as well as a final incubation in toluol (Carl Roth #7115.1) for 5 min, the cryosections were air-dried at RT and embedded using the mounting medium DPX (Sigma Aldrich #1.00579). To quantify CM cross-sectional areas, stained cryosections were imaged using a histoscanner (Pannoramic® Flash DESK DX, 3DHISTECH) and the sizes of 50 randomly chosen CMs with a centrally located nucleus were determined by ImageJ (ImageJ 1.53).

The extent of Ang II-induced cardiac fibrosis development was determined by picrosirius red (PSR) staining of cardiac cryosections derived from untreated or Ang II treated CTR and *cmf*KO mice as previously described (2, 8). Thawed cryosections selected from 8 distinct regions of each heart were fixed in Boiun’s solution (Sigma Aldrich #HT101126) for over 24 h before samples were incubated in Sirius Red staining solution (0.1% Direct Red 80 dissolves in aqueous saturated picric acid) for 1 h to specifically visualize collagen fibers. Excess dye solution was removed by multiple washing steps in 0.01 N HCl and ddH_2_O. Upon dehydration of the samples with increasing concentrations of ethanol (50%, 70%, 90%, 100%) and lastly with 100% xylene, samples were air-dried at RT and mounted in non-aqueous DPX. Heart sections were imaged using a histoscanner (Pannoramic® Flash DESK DX, 3DHISTECH), and collagen deposition (stained red) was analyzed with ImageJ (ImageJ 1.53) as a percentage of the total heart area of the selective section.

A commercial alkaline phosphatase staining kit (Vectastain ABC-AP kit Alkaline Phosphatase (Standard), Vector laboratories #AK 5000) was used to detect the expression of specific target proteins in fibrotic areas of hearts obtained from Ang II-treated CTR and *cmf*KO mice as described previously (9). Consistent with previously published studies thawed cardiac cryosections were permeabilized by 0.3% Triton X-100 in DPBS for 30 min and washed several times with DPBS (6, 8). To prevent unspecific binding of the primary antibody, samples were incubated for 1 h at RT in blocking buffer (DPBS supplemented with 10% normal goat serum (NGS)) before being loaded overnight at 4°C with primary antibody against either periostin (1:500, Abcam #ab14041) or cGKI (1:1000, Cell signaling Technology #3248S) diluted in 1.5% NGS in DPBS. After multiple washing steps with 1.5% NGS in DPBS, samples were incubated with biotinylated secondary antibodies (Goat Anti-Rabbit IgG (H+L) biotinylated 1:1000; Vector laboratories #BA-1000) for 1 h at RT. According to the manufacturer’s recommendations, APC-AP reagent (5 ml DPBS with one drop Reagent A and B) was applied to the sections for 30 min in the dark followed by several wash steps in DPBS and a final incubation of the samples in AP-substrate (5 ml 100 mM Tris-HCL pH 8.2 with Reagent 1, 2 and 3 as well as Levamisole, Vector Laboratories #SK5300 and #SP-5000-18) in order to develop the staining. After 10-20 min phosphatase activity was stopped by rising the samples in tap water. The stained samples were air-dried, embedded in the aqueous mounting medium Aquatex® (Merck Millipore #108562) and visualized using a histoscanner (Pannoramic® Flash DESK DX, 3DHISTECH).

## Nuclear staining of CFs/CMFs with Hoechst

Isolated CFs/CMFs were cultured *in vitro* on coverslips (Marienfeld Superior #0111580) for five days and were then fixed with 4% PFA for 10 min. After washing thrice with DPBS, cells were permeabilized with 0.3% Triton X-100 for 15 min at RT. After renewed multiple washing steps with DPBS, nuclei staining was performed by embedding the cells with 0.1% Hoechst 33432 (Thermo Scientific #62249) in PermaFluor aqueous mounting media (Thermo Scientific #TA-030-FM) and detected using a fluorescent Apotome microscope (Zeiss).

**WGA staining**

Thawed heart cryosections from CTR and cmfKO animals were stained with fluorescently labelled Wheat Germ Agglutinin (WGA) (W32464, Invitrogen™) to analyze the CM cross-sectional area. By applying a chemical cocktail consisting of 3% H_2_O_2_ and 50 mM NaOH for 45 min, autofluorescence of cardiac cryosections was diminished (4). After three washing steps with DPBS, WGA staining solution (5 µg/ml in DPBS) was added for 20 min at RT. Followed by three washing steps with DPBS, nuclei were stained with Hoechst 33432 (1:1000) for 10 min at RT and sections were mounted in PermaFluor aqueous mounting media. To quantify CM cross-sectional areas, stained cryosections were imaged using fluorescent Apotome microscope (Zeiss) and the sizes of randomly chosen CMs were determined by ImageJ (ImageJ 1.53).

## DNA extraction and PCR analysis

Allele-specific polymerase chain reaction (PCR) was performed to demonstrate Cre-mediated CMF-specific ablation of the floxed cGKI gene locus. For control organs (brain, liver, kidney, spleen, lung, aorta) and CF/CMF primary cells isolated as outlined before, DNA extraction was performed according to the manufacturer's instructions using a High Pure PCR Template Preparation Kit (Roche #11796828001). Amplification of the DNA was achieved by employing the KAPA HotStart Mice-Genotyping Kit (Roche #KK7352). Cre-mediated excision of the floxed exon 10 of *Prkg1,* encoding for cGKI, was confirmed by the use of three distinct primers ((1) cGKI_forward 5’-CCT GGC TGT GAT TTC ACT CCA-3’; (2) cGKI_reverse1 5’-AAA TTA TAA CTT GTC AAA TTC TTG-3’; (3) cGKI_reverse2 5’-GTC AAG TGA CCA CTA TG-3’) as previously described (10).

## Western Blot analysis

Western Blot analyses were conducted with CF/CMF protein lysates obtained from CTR and *cmf*KO mice following 28 days of Ang II treatment *in vivo*. Isolated CFs/CMFs were cultured *in vitro* until reaching 90% confluency and then suspended in lysis buffer (ddH_2_O containing 50 mM Tris pH 7.4, 150 mM NaCl, 10% Glycerol, 2 mM EDTA, 10 mM NaPyrophosphate, 2.5 mM NaF, 200 µM Na_3_VO_4_, 1% NP-40, 2.5 mM MgCl_2_). To study the 8-Br-cGMP-dependent phosphorylation of the vasodilator-stimulated phosphoprotein (pVASP) cells at 90% confluency were starved for 24 h with FBS-free culture medium and were then restimulated with 8-Bromo-cGMP (1 mM) for 24h and then suspended in lysis buffer.

For the adjustment of the total protein concentration to 40 µg, the BCA assay (Thermo Scientific #23227) was employed according to the manufacturer’s instructions. The total protein amount was subsequently incubated in 4x Laemmli sample buffer (62.5 mM Tris-HCl pH 6.9, 10% Glycerol, 1 %SDS, 0.0005 % bromophenol blue) at 95°C for 10 min, prior to separation of the proteins by gel electrophoresis (80-120 V) in 12.5% polyacrylamide gel. The protein marker IV (Peqlab peqGOLD, VWR #27-2110) was used for estimating the molecular weight of distinct proteins. By using a semi-dry blotting system (Carl Roth, 60 mA for 1 h following 90 mA for 15 min), the proteins were transferred to polyvinylidene fluoride (PVDF) membranes (Merck Millipore #IPFL00010). Upon blocking unspecific binding sites of the membranes using 5% dry milk in 20 mM Tris-buffered saline containing 0.05 % Tween 20 for 1 h at RT, the membranes were incubated in the primary antibody solution (cGKI 1:250 dilution, Cell signaling Technology #3248S; GAPDH 1:1000 dilution, Cell signaling Technology #2118S) at 4 °C overnight. Subsequently a horseradish peroxidase-coupled secondary antibody (rabbit IgG HRP-linked whole Ab from donkey, 1:1000 dilution, Merck Millipore #GENA934) was applied for the detection of the specific primary antibody binding. Finally, the immunosignals were visualized after a 30 sec incubation of the membranes in the chemiluminescent substrate (ECL Western Blotting Substrate, Merck Millipore #WBLUF0100) using an Amersham Imager 600 (General Electrics) and analyzed densitometrically with ImageJ (ImageJ 1.53).

**RT-qPCR**

Isolated CFs/CMFs were cultured *in vitro* for 7-10 days until reaching 90% confluency, starved for 24 h with FBS-free culture medium and stimulated with 8-Bromo-cGMP (1 mM) for 24h. RNA was isolated according to manufacturer’s instructions using NucleoSpin™ Isolation-Kit (740955, Macherey-Nagel™). Isolated RNA was eluted with 60 µl DEPC water (T143.5, Carl Roth). Reverse transcription for cDNA synthesis was performed according to manufacturer’s instructions using iScript™ cDNA Synthesis Kit (1708891, Bio-Rad) and 0.5 µg of the isolated RNA. qRT-PCR was performed with the CFX Connect Real-Time PCR Detection System (Bio-Rad) using SsoAdvanced Universal SYBR® Green Supermix (1725274, Bio-Rad) and specific primer pairs.

## Grid-based proliferation assay

Grid-based proliferation assay was employed to assess the proliferation behavior of primary CF/CMF cell cultures isolated from Ang II treated CTR and *cmf*KO mice as previously described (11). A cell suspension adjusted to 20,000 cells was seeded into each well of an 8-well Grid-500 µm slide (Ibidi #80806-G500). Cells were allowed to adhere to the bottom of the slide for 24 h at 5 % CO_2_ and 37°C, prior to starvation (DMEM + GlutaMAX^TM^ supplemented with 1 % PenStrep) for another 24 h to synchronize the cell cycle of the cells. Immediately after the addition of culture medium (time point t = 0 h) and for five consecutive days (at intervals of 24 h), images of four selected quadrants within each well of the Grid-slide were acquired using an inverted transmitted-light microscope (Zeiss Axiovert 200M) to monitor proliferation rate. For quantification, the average of the total cell number from three different wells was calculated, with the total cell number of one well resulting from the mean of the four observed squares.

## Supplemental Reagents and Tools

| **Reagent/Resource** | **Reference or Source** | **Identifier or Catalog Number** |
| --- | --- | --- |
| **Experimental Models** |  |  |
| Mouse: Tg(Postnicre/ERT2)#Wet | Kaur et al., 2016 | N/A |
| Mouse: PRKG1^tm2Naw^ | Wegener et al., 2002 | N/A |
| Mouse:(*B6.129(Cg)Gt(ROSA)26Sor^tm4(ACTB-tdTomato,-EGFP)Luo^/J*) | Charles River | Stock No. 007576, Jackson Laboratory |
| **Antibodies** |  |  |
| Goat anti-Mouse IgG1 Alexa Fluor 488 1:2000 | Thermo Scientific | Cat#A-21121 |
| Goat anti-Rabbit (H+L) Alexa Fluor 555 1:1000 | Thermo Scientific | Cat#A-21428 |
| Goat anti-Rabbit (H+L) Alexa Fluor 647 1:1000 | Thermo Scientific | Cat#A32733 |
| Goat anti-Rabbit (H+L) biotinylated | Vector laboratories | Cat#BA-1000 |
| Mouse monoclonal Anti-Collagen I (1:1000) | Abcam | Cat#ab6308 |
| Rabbit IgG HRP-linked whole Ab (from donkey) (1:1000 WB) | Merck Millipore | Cat#GENA934 |
| Rabbit IgG PKG-1 (1:1000 IHC, 1:250 WB) | Cell Signaling Technology | Cat#3248S |
| Rabbit monoclonal Anti-Collagen III (1:500) | Abcam | Cat#ab184993 |
| Rabbit monoclonal GAPDH (1:1000) | Cell Signaling Technology | Cat#2118S |
| Rabbit monoclonal Ki67 (1:1000) | Cell Signaling Technology | Cat#9129 |
| Rabbit polyclonal Anti-Periostin (1:500) | Abcam | Cat#ab14041 |
| Rabbit polyclonal Anti-VASP (1:1000) | Cell Signaling Technology | Cat##3132 |
| Rabbit polyclonal Anti-phospho-VASP (1:1000) | Cell Signaling Technology | Cat#3114 |
| **Oligonucleotides and other sequence-based reagents** |  |  |
| PostniCre 3HA-forward 5’-CAT GCT GGG GAT GCG GTG GG-3’ | Kaur et al., 2016 |  |
| PostniCre 3HA-reverse 5’-CTT GCA GAA CAG TGG CCT GGG A-3’ | Kaur et al., 2016 |  |
| PostniCre 5HA-forward 5’-TGC CCC TGT GAT TTC TCT TC-3’ | Kaur et al., 2016 |  |
| PostniCre 5HA-reverse 5’-GGA GCA TCT TCC AGG TGT GT-3’ | Kaur et al., 2016 |  |
| cGKI forward 5’-CCT GGC TGT GAT TTC ACT CCA-3’ | Wegener et al., 2001 |  |
| cGKI reverse1 5’-AAA TTA TAA CTT GTC AAA TTC TTG-3’ | Wegener et al., 2001 |  |
| cGKI reverse2 5’-GTC AAG TGA CCA CTA TG-3’ | Wegener et al., 2001 |  |
| **Primers for qRT-PCR analysis** | Sequence 5’to 3‘ |  |
| Tnni forward | AGA GCT TCA GGA CTT ATG CCG ACA | |
| Tnni reverse | TGG TGA CTT TTG CTT CCA CGT C | |
| Icam1 forward | CGG ACT TTC GAT CTT CCA GCT ACC | |
| Icam1 reverse | CGA GCT TCA GAG GCA GGA AAC A | |
| Pecam forward | TTG CAG TCA GAG TCT TCC TTG CC | |
| Pecam reverse | GGG TTT CTG TTT GGC CTT GGC T | |
| Acta2 forward | AGA GGC ACC ACT GAA CCC TA | |
| Acta2 reverse | GCA TAG AGG GAC AGC ACA GC | |
| Col1a1 forward | AGG ATC TCC TGG TGC TGA TGG A | |
| Col1a1 reverse | TTT GCC AGG TTC ACC AGA GGG | |
| Fn1 forward | CGG AGA GAG TGC CCC TAC TAC | |
| Fn1 reverse | GAC CCG CAT CGT GTC TGG A | |
| IL6 forward | CGA TGA TGC ACT TGC AGA AA | |
| IL6 reverse | ACT CCA GAA GAC CAG AGG AA | |
| Tgfb1 forward | TGC TAA TGG TGG ACC GCA AC | |
| Tgfb1 reverse | GCA CTG CTT CCC GAAT GTC TG | |
| Hprt forward | CGC AGT CCC AGC GTC GTG ATT A | |
| Hprt reverse | CGA GCA AGT CTT TCA GTC CTG TCC | |
| **Chemicals, Enzymes, and other reagents** |  |  |
| Acetic acid (100%) | Carl Roth | Cat#3738.5 |
| Ammonia (30-33%) | Carl Roth | P093.2 |
| Angiotensin II human | Sigma-Aldrich | Cat#A9525 |
| Aquatex® | Merck Millipore | Cat#108562 |
| Boiun solution | Sigma-Aldrich | Cat#HT101126 |
| Bovine serum albumin (BSA) | Carl Roth | Cat#8076 |
| Bromophenol blue | Serva | 15375 |
| CLS-2 – Collagenase, Type 2 | Cellsystems® | Cat#LS004177 |
| DEPC Water | Carl Roth | Cat# T143.5 |
| Direct Red 80 | Sigma-Aldrich | Cat#365548 |
| DMEM + GlutaMAX^TM^ | Thermo Scientific | Cat#31966021 |
| DPX | Sigma-Aldrich | Cat#1.00579 |
| Dry milk powder | Carl Roth | Cat#T145.3 |
| Dulbecco's Phosphate Buffered Saline (DPBS) | Thermo Scientific | Cat#14190094 |
| ECL Western Blotting Substrate | Merck Millipore | Cat##WBLUF0100 |
| EDTA | Carl Roth | Cat#8043.1 |
| Eosin-G solution (0.5 %) | Carl Roth | Cat#X883.1 |
| Fetal bovine serum (FBS) | Thermo Scientific | Cat#26140079 |
| Glycerol | Carl Roth | Cat#3738.1 |
| H_2_O_2_ | Carl Roth | Cat#8070.2 |
| Harris Haematoxylin solution | Carl Roth | X903.2 |
| HCl (0.1N) | Carl Roth | Cat#6789.1 |
| HEPES | Carl Roth | Cat#9105.3 |
| Hoechst 33432 | Thermo Scientific | Cat#62249 |
| Insulin-Transferrin-Selen (ITS-G) (100X) | Thermo Scientific | Cat#41400045 |
| iScript™ cDNA Synthesis Kit | Bio-Rad | Cat#1708891 |
| Isotonic NaCl-solution 0.9 % | DELTAMEDICA | PZ16497/03 |
| KCl | Carl Roth | Cat#6781.1 |
| L-Glutamic acid Monosodium Salt | Sigma-Aldrich | Cat#G-1626 |
| Levamisole solution | Vector laboratories | Cat#SP-5000-18 |
| Magnesium chloride hexahydrate (MgCl_2_*6H_2_O) | Merck Millipore | Cat#1.05833 |
| MgCl_2_ | Carl Roth | Cat#KK36.3 |
| Miglyol®812 | Caelo | Cat#3274 |
| Na_3_VO_4_ | Carl Roth | Cat#0735.1 |
| NaCl | Carl Roth | Cat#3957.2 |
| NaF | Carl Roth | Cat#2618.1 |
| NaOH | Carl Roth | Cat#6771 |
| NaPyrophosphate | Sigma-Aldrich | Cat#P8010 |
| Neg-50^TM^ | Epredia | Cat#6502 |
| NH_4_Cl | Carl Roth | Cat#5470.1 |
| Normal goat serum (NGS) | BIOZOL Diagnostica | Cat#ENG9010 |
| NP-40 | Sigma-Aldrich | Cat#I3021 |
| NucleoSpin™ Isolation-Kit | Macherey-Nagel | Cat#740955 |
| Paraformaldehyde | Carl Roth | Cat#0335.3 |
| Penicillin-Streptomycin (10,000 U/ml) | Thermo Scientific | Cat#15140122 |
| PermaFluor Aqueous Mounting Medium | Thermo Scientific | Cat#TA-030-FM |
| Picric acid solution (saturated) | Sigma-Aldrich | Cat#6744-1GA |
| Protein marker IV | VWR | Cat#27-2110 |
| SsoAdvanced Universal SYBR® Green Supermix | Bio-Rad | Cat#1725274 |
| Sodium dodecyl sulfate pellets (SDS) | Carl Roth | Cat#8029.3 |
| Tamoxifen | Sigma-Aldrich | Cat#T5648 |
| Toluol | Carl Roth | Cat#7115.1 |
| Tris | Carl Roth | Cat#3051 |
| Triton-X-100 | Carl Roth | Cat#3051 |
| Tween-20 | Carl Roth | Cat#9127.1 |
| Vectastain ABC-AP kit Alkaline Phosphatase (Standard) | Vector laboratories | Cat#AK 5000 |
| Vector® Blue Substrate Kit, Alkaline Phsophatase (AP) | Vector laboratories | Cat#SK5300 |
| Wheat Germ Agglutinin | Invitrogen | Cat#W32464 |
| Xylene | Carl Roth | Cat#9713.3 |
| **Software** |  |  |
| Dataquest A.R.T.3.1 | Data Sciences International | https://www.datasci.com/products/software/dataquest-art |
| Excel Office 365 | Microsoft | https://www.office.com |
| ImageJ 1.53v | NIH | https://imagej.net/ij/index.html |
| Prism 9.4.1 | GraphPad | https://www.graphpad.com/ |
| VevoStrain | VisualSonics | https://www.visualsonics.com/product/software/vevo-strain-software |
| Zen Lite 2.6 | Zeiss | https://www.zeiss.de/mikroskopie/produkte/mikroskopsoftware/zen-lite.html |
| **Other** |  |  |
| ALZET® pump model 1004 | ALZET | Cat#0009922 |
| BCA assay | Thermo Scientific | Cat#23227 |
| High Pure PCR Template Preparation-Kit | Roche | Cat#11796828001 |
| KAPA2G Fast HotStart Mice Genotyping Kit | Roche | Cat#KK7352 |

**References**

Table S1: **Values and statistics for Figure 1.**

| **Panel** |  | | | | | | | |  | | | |
| --- | --- | --- | --- | --- | --- | --- | --- | --- | --- | --- | --- | --- |
| **C** | **genotype** | | **relative cGKI expression** | | | | **n** | **statistics** | | | **p** | |
|  | CTR | | 0.84 ± 0.15 | | | | 6 | unpaired t-test | | |  | |
|  | *cmf*KO | | 0.43 ± 0.05 | | | | 6 |  |  |  | ***** = 0.02 *vs.* CTR | |
|  |  | |  | | | |  |  | | |  | |
| **E** | **genotype** | | **treatment** | | **survival** | | **n** | **statistics** | | | **p** | |
|  | CTR | | +TAM | | 100 % | | 13 | Log-rank (Mantel-Cox) test | | |  | |
|  | CTR | | + Tam  +Ang II | | 84,6 % | | 39 |  |  |  | 0.14 *vs.* CTR+TAM | |
|  | *cmf*KO | | +TAM | | 100 % | | 12 | Log-rank (Mantel-Cox) test | | |  | |
|  | *cmf*KO | | + Tam  +Ang II | | 71,4 % | | 42 |  |  |  | **†** = 0.045 *vs.* *cmf*KO+TAM | |
|  | CTR | | +TAM | | 100 % | | 13 | Log-rank (Mantel-Cox) test | | |  | |
|  | *cmf*KO | | +TAM | | 100 % | | 12 |  |  |  | >0.9999 *vs.* CTR+TAM | |
|  | CTR | | + Tam  +Ang II | | 84,6 % | | 39 | Log-rank (Mantel-Cox) test | | |  | |
|  | *cmf*KO | | + Tam  +Ang II | | 71,4 % | | 42 |  |  |  | 0.14 *vs.* CTR+TAM+Ang II | |
|  | |  | |  | |  | | | |  | |  |
| **F** | **genotype** | | **Ang II [days]** | | **MAP [mmHg]** | | **n** | **statistics** | | | **p** | |
|  | CTR | | **0** | | 112.51 ± 4.11 | | 4 | 2way ANOVA  F_14,90_ = 3.3  **#p = 0.0003**  **(time [days])**  2way ANOVA  F_1,90_ = 3.3  p = 0.07  (**genotype**) | | |  | |
|  | *cmf*KO | |  |  | 110.50 ± 1.30 | | 4 |  |  |  | >0.99 *vs.* CTR | |
|  | CTR | | **0.5** | | 129.00 ± 4.76 | | 4 |  |  |  |  | |
|  | *cmf*KO | |  |  | 116.21 ± 4.21 | | 4 |  |  |  | 0.83 *vs.* CTR | |
|  | CTR | | **1** | | 128.41 ± 6.02 | | 4 |  |  |  |  | |
|  | *cmf*KO | |  |  | 117.32 ± 4.81 | | 4 |  |  |  | 0.93 *vs.* CTR | |
|  | CTR | | **1.5** | | 128.37 ± 8.04 | | 4 |  |  |  |  | |
|  | *cmf*KO | |  |  | 122.15 ± 3.34 | | 4 |  |  |  | 0.99 *vs.* CTR | |
|  | CTR | | **2** | | 136.80 ± 5.62 | | 4 |  |  |  |  | |
|  | *cmf*KO | |  |  | 141.37 ± 6.45 | | 4 |  |  |  | >0.99 *vs.* CTR | |
|  | CTR | | **2.5** | | 127.77 ± 7.86 | | 4 |  |  |  |  | |
|  | *cmf*KO | |  |  | 136.43 ± 2.31 | | 4 |  |  |  | 0.99 *vs.* CTR | |
|  | CTR | | **3** | | 136.00 ± 7.79 | | 4 |  |  |  |  | |
|  | *cmf*KO | |  |  | 138.36 ± 3.76 | | 4 |  |  |  | >0.99 *vs.* CTR | |
|  | CTR | | **3.5** | | 132.55 ± 8.53 | | 4 |  |  |  |  | |
|  | *cmf*KO | |  |  | 131.59 ± 7.86 | | 4 |  |  |  | >0.99 *vs.* CTR | |
|  | CTR | | **4** | | 137.95 ± 7.43 | | 4 |  |  |  |  | |
|  | *cmf*KO | |  |  | 130.53 ± 5.18 | | 4 |  |  |  | 0.99 *vs.* CTR | |
|  | CTR | | **4.5** | | 135.33 ± 7.00 | | 4 |  |  |  |  | |
|  | *cmf*KO | |  |  | 127.26 ± 5.23 | | 4 |  |  |  | 0.99 *vs.* CTR | |
|  | CTR | | **5** | | 137.98 ± 5.53 | | 4 |  |  |  |  | |
|  | *cmf*KO | |  |  | 127.67 ± 8.30 | | 4 |  |  |  | 0.99 *vs.* CTR | |
|  | CTR | | **5.5** | | 132.42 ± 4.34 | | 4 |  |  |  |  | |
|  | *cmf*KO | |  |  | 129.89 ± 6.53 | | 4 |  |  |  | >0.99 *vs.* CTR | |
|  | CTR | | **6** | | 138.70 ± 4.09 | | 4 |  |  |  |  | |
|  | *cmf*KO | |  |  | 129.37 ± 5.12 | | 4 |  |  |  | 0.98 *vs.* CTR | |
|  | CTR | | **6.5** | | 135.63 ± 3.43 | | 4 |  |  |  |  | |
|  | *cmf*KO | |  |  | 133.38 ± 2.28 | | 4 |  |  |  | >0.99 *vs.* CTR | |
|  | CTR | | **7** | | 136.23 ± 4.43 | | 4 |  |  |  |  | |
|  | *cmf*KO | |  |  | 137.61 ± 1.30 | | 4 |  |  |  | >0.99 *vs.* CTR | |
|  | | | | | | | | | | | | |
| **G** | **genotype** | | **MAP [mmHg]** | | | | **n** | **statistics** | | | **p** | |
|  | CTR | | 27.37 ± 4.04 | | | | 4 | Multiple unpaired t-test | | |  | |
|  | *cmf*KO | | 23.02 ± 4.00 | | | | 4 |  |  |  | 0.47 *vs.* CTR | |
|  | **genotype** | | **Systolic Pressure [mmHg]** | | | | **n** |  |  |  | **p** | |
|  | CTR | | 34.00 ± 5.00 | | | | 4 |  |  |  |  | |
|  | *cmf*KO | | 28.87 ± 6.61 | | | | 4 |  |  |  | 0.56 *vs.* CTR | |
|  | **genotype** | | **Diastolic Pressure [mmHg]** | | | | **n** |  |  |  | **p** | |
|  | CTR | | 21.99 ± 5.25 | | | | 4 |  |  |  |  | |
|  | *cmf*KO | | 20.61 ± 3.05 | | | | 4 |  |  |  | 0.83 *vs.* CTR | |
|  | **genotype** | | **Pulse pressure [mmHg]** | | | | **n** |  |  |  | **p** | |
|  | CTR | | 12.18 ± 3.95 | | | | 4 |  |  |  |  | |
|  | *cmf*KO | | 8.28 ± 5.83 | | | | 4 |  |  |  | 0.60 *vs.* CTR | |

Table S2: **Values and statistics for Figure 2**

| **Panel** |  | | | |  | | | |
| --- | --- | --- | --- | --- | --- | --- | --- | --- |
| **B** | **genotype** | **treatment** | | **amount of fibrosis**  [% of total heart] | | **n** | **statistics** | **p** |
|  | CTR | +TAM | | 2.20 ± 0.19 | | 8 | 2way ANOVA  F_1,29_ = 5.43  p = 0.03  (**genotype**)  2way ANOVA  F_1,29_ = 29.75  **p = <0.0001**  (**treatment**) |  |
|  | *cmf*KO |  |  | 2.61 ± 0.18 | | 8 |  | 0.79 *vs.* CTR+TAM |
|  | CTR | +TAM + Ang II | | 5.80 ± 0.82 | | 8 |  | **†** = 0.02 *vs.* CTR+TAM |
|  | *cmf*KO |  |  | 10.14 ± 1.69 | | 9 |  | **#** <0.0001 *vs.* *cmf*KO+TAM  ****** = 0.005 *vs.* CTR+TAM+Ang II |
|  | | | | | | | | |
| **C** | **genotype** | **heart section** | **treatment** | **collagen deposition**  [% of cardiac section] | | **n** | **statistics** | **p** |
|  | CTR | **I** | +TAM | 3.37 ± 0.52 | | 8 | 2way ANOVA  F_3,227_ = 60,84  **p < 0.0001**  (**genotype; treatment**)  2way ANOVA  F_7,227_ = 0.25  p = 0.97  (**segments**) |  |
|  | *cmf*KO |  |  | 3.87 ± 0.41 | | 8 |  | 0.80 *vs.* CTR+TAM |
|  | CTR |  | +TAM  + Ang II | 5.30 ± 0.75 | | 8 |  | 0.33 *vs.* CTR+TAM |
|  | *cmf*KO |  |  | 9.34 ± 2.11 | | 9 |  | **§** = 0.005 *vs.* *cmf*KO+TAM  0.04 *vs.* CTR+TAM+Ang II (q-value >0.05) |
|  | CTR | **II** | +TAM | 2.51 ± 0.54 | | 8 |  |  |
|  | *cmf*KO |  |  | 2.57 ± 0.41 | | 8 |  | 0.97 *vs.* CTR+TAM |
|  | CTR |  | +TAM  + Ang II | 4.92 ± 0.97 | | 8 |  | 0.23 *vs.* CTR+TAM |
|  | *cmf*KO |  |  | 9.32 ± 2.30 | | 9 |  | **#** < 0.0006 *vs.* *cmf*KO+TAM  *****= 0.03 *vs.* CTR+TAM+Ang II |
|  | CTR | **III** | +TAM | 1.67 ± 0.23 | | 8 |  |  |
|  | *cmf*KO |  |  | 2.03 ± 0.24 | | 8 |  | 0.86 *vs.* CTR+TAM |
|  | CTR |  | +TAM  + Ang II | 5.64 ± 1.66 | | 8 |  | **†** = 0.049*vs.* CTR+TAM |
|  | *cmf*KO |  |  | 10.40 ± 2.28 | | 9 |  | **#** < 0.0001 *vs.* *cmf*KO+TAM  *****= 0.02 *vs.* CTR+TAM+Ang II |
|  | CTR | **IV** | +TAM | 1.91 ± 0.35 | | 8 |  |  |
|  | *cmf*KO |  |  | 2.55 ± 0.43 | | 8 |  | 0.75 *vs.* CTR+TAM |
|  | CTR |  | +TAM  + Ang II | 6.29 ± 1.41 | | 8 |  | **†** = 0.03 *vs.* CTR+TAM |
|  | *cmf*KO |  |  | 11.88 ± 3.05 | | 9 |  | **#** < 0.0001 *vs.* *cmf*KO+TAM  ****** = 0.005 *vs.* CTR+TAM+Ang II |
|  | CTR | **V** | +TAM | 1.84 ± 0.23 | | 8 |  |  |
|  | *cmf*KO |  |  | 2.03 ± 0.20 | | 8 |  | 0.93 *vs.* CTR+TAM |
|  | CTR |  | +TAM  + Ang II | 6.92 ± 1.62 | | 8 |  | **†** = 0.01 *vs.* CTR+TAM |
|  | *cmf*KO |  |  | 11.29 ± 2.52 | | 9 |  | **#** < 0.0001 *vs.* *cmf*KO+TAM  ***** = 0.03 *vs.* CTR+TAM+Ang II |
|  | CTR | **VI** | +TAM | 1.35 ± 0.11 | | 8 |  |  |
|  | *cmf*KO |  |  | 2.23 ± 0.25 | | 8 |  | 0.66 *vs.* CTR+TAM |
|  | CTR |  | +TAM  + Ang II | 6.10 ± 1.18 | | 8 |  | **†** = 0.02 *vs.* CTR+TAM |
|  | *cmf*KO |  |  | 10.27 ± 2.15 | | 9 |  | **#** < 0.0001 *vs.* *cmf*KO+TAM  ***** = 0.03 *vs.* CTR+TAM+Ang II |
|  | CTR | **VII** | +TAM | 1.70 ± 0.21 | | 8 |  |  |
|  | *cmf*KO |  |  | 1.95 ± 0.16 | | 8 |  | 0.90 *vs.* CTR+TAM |
|  | CTR |  | +TAM  + Ang II | 6.17 ± 1.08 | | 7 |  | **†** = 0.03 *vs.* CTR+TAM |
|  | *cmf*KO |  |  | 9.65 ± 1.81 | | 9 |  | **§** = 0.0001 *vs.* *cmf*KO+TAM  0.09 *vs.* CTR+TAM+Ang II |
|  | CTR | **VIII** | +TAM | 1.53 ± 0.17 | | 8 |  |  |
|  | *cmf*KO |  |  | 2.32 ± 0.20 | | 8 |  | 0.69 *vs.* CTR+TAM |
|  | CTR |  | +TAM  + Ang II | 5.76 ± 1.13 | | 6 |  | 0.05 *vs.* CTR+TAM |
|  | *cmf*KO |  |  | 9.69 ± 1.74 | | 7 |  | **§** = 0.0005 *vs.* *cmf*KO+TAM  0.08 *vs.* CTR+TAM+Ang II |
|  | | | | | | | | |
| **E** | **genotype** | **treatment** | | **HW** [mg] | | **n** | **statistics** | **p** |
|  | CTR | +TAM | | 150.41 ± 4.90 | | 10 | 2way ANOVA  F_1,32_ = 19.57  **p < 0.0001**  **(treatment)**  2way ANOVA  F_1,32_ = 0.04  p = 0.85  (genotype) |  |
|  | *cmf*KO |  |  | 148.58 ± 5.93 | | 9 |  | 0.99 *vs.* CTR+TAM |
|  | CTR | +TAM + Ang II | | 181.41 ± 11.78 | | 8 |  | **†** = 0.04 *vs.* CTR+TAM |
|  | *cmf*KO |  |  | 186.21 ± 8.13 | | 9 |  | **§** = 0.008 *vs.* *cmf*KO+TAM; |
|  |  |  |  |  |  |  |  | 0.97 vs CTR+TAM+Ang II |
|  | | | | | | | | |
| **F** | **genotype** | **treatment** | | **HW/TL** [mg/mm] | | **n** | **statistics** | **p** |
|  | CTR | +TAM | | 8.42 ± 0.24 | | 10 | 2way ANOVA  F_1,32_ = 27.05  **p < 0.0001**  **(treatment)**  2way ANOVA  F_1,32_ = 0.647  p = 0.43  (**genotype**) |  |
|  | *cmf*KO |  |  | 8.43 ± 0.34 | | 9 |  | >0.9999 *vs.* CTR+TAM |
|  | CTR | +TAM + Ang II | | 10.31 ± 0.63 | | 8 |  | **†** = 0.02 *vs.* CTR+TAM |
|  | *cmf*KO |  |  | 11.00 ± 0.49 | | 9 |  | **#** = 0.0009 *vs.* *cmf*KO+TAM |
|  |  |  |  |  |  |  |  | 0.69 vs CTR+TAM+Ang II |
|  | | | | | | | | |
| **G** | **genotype** | **HW/TL** [%] | | | | **n** | **statistics** | **p** |
|  | CTR | 22.43 ± 7.41 | | | | 8 | unpaired t-test |  |
|  | *cmf*KO | 30.53 ± 5.80 | | | | 9 |  | 0.40 *vs.* CTR |

| **I** | **genotype** | **treatment** | **CM-cross sectional area** [µm^2^] | **n** | **statistics** | **p** |
| --- | --- | --- | --- | --- | --- | --- |
|  | CTR | +TAM | 131.35 ± 4.81 | 8 | 2way ANOVA  F_1,26_ = 133.6  **p < 0.0001**  (**treatment**)  2way ANOVA  F_1,26_ = 3.266  p = 0.08 |  |
|  | *cmf*KO |  | 123.67 ± 7.04 | 6 |  | 0.90 *vs.*  CTR+TAM |
|  | CTR | +TAM + Ang II | 200.61 ± 11.92 | 7 |  | **#** <0.0001 *vs.*  CTR+TAM |
|  | *cmf*KO |  | 236.80 ± 6.57 | 9 |  | **#** <0.0001 *vs.* *cmf*KO+TAM; |
|  |  |  |  |  |  | ***** = 0.01 vs CTR+TAM+Ang II |

**Table S3: Values and statistics for Figure** 3

| **Panel** |  | | |  | | |
| --- | --- | --- | --- | --- | --- | --- |
| **B** | **genotype** | **TUNEL^+^ CMs per 0.15 mm^2^**  [% of total CMs] | **n** | | **statistics** | **p** |
|  | CTR | 5.17 ± 1.63 | 5 | | unpaired t-test |  |
|  | *cmf*KO | 17.34 ± 4.40 | 6 | |  | ***** = 0.04 *vs.* CTR |
|  | | | | | | |
| **C** | **genotype** | **TUNEL^+^ CMs per 0.15 mm^2^**  [% of total CMs] | **n** | | **statistics** | **p** |
|  | CTR | 4.79 ± 1.16 | 6 | | unpaired t-test |  |
|  | *cmf*KO | 7.28 ± 1.76 | 6 | |  | 0.27 *vs.* CTR |
|  | | | | | | |
| **D** | **genotype** | **TUNEL^+^ CMs per 0.15 mm^2^**  [% of total CMs] | **n** | | **statistics** | **p** |
|  | CTR | 4.96 ± 0.92 | 11 | | unpaired t-test |  |
|  | *cmf*KO | 12.31 ± 2.72 | 12 | |  | *****= 0.02 *vs.* CTR |
|  | | | | | | |

Table S4: **Values and statistics for Figure 4**

| **Panel** |  | | | | |  | | |
| --- | --- | --- | --- | --- | --- | --- | --- | --- |
| **B** | **genotype** | **Ki67^+^ nuclei per 0.15 mm^2^** | | | **n** | | **statistics** | **p** |
|  | CTR | 0.93 ± 0.31 | | | 8 | | unpaired t-test |  |
|  | *cmf*KO | 3.00 ± 0.46 | | | 9 | |  | ****** = 0.003 *vs.* CTR |
|  | | | | | | | | |
| **D** | **genotype** | **time [h]** | | **relative proliferation rate** | **n** | | **statistics** | **p** |
|  | CTR | **0** | | 1.00 ± 0.00 | 8 | | 2way ANOVA  F_5,71_ = 21.66  p < 0.0001  **(time [h])**  2way ANOVA  F_1,71_ =16.47  p = 0.0001  **(genotype)** |  |
|  | *cmf*KO |  |  | 1.00 ± 0.00 | 6 | |  | >0.9999 *vs.* CTR |
|  | CTR | **24** | | 0.93 ± 0.11 | 8 | |  |  |
|  | *cmf*KO |  |  | 1.15 ± 0.06 | 6 | |  | 0.99 *vs.* CTR |
|  | CTR | **48** | | 1.57 ± 0.18 | 8 | |  |  |
|  | *cmf*KO |  |  | 2.42 ± 0.28 | 6 | |  | 0.94 *vs.* CTR |
|  | CTR | **72** | | 2.27 ± 0.32 | 8 | |  |  |
|  | *cmf*KO |  |  | 4.84 ± 1.04 | 6 | |  | 0.05 *vs.* CTR |
|  | CTR | **96** | | 3.77 ± 0.79 | 7 | |  |  |
|  | *cmf*KO |  |  | - 1. 1.24 | 6 | |  | *****= 0.03 *vs.* CTR |
|  | CTR | **120** | | 4.81 ± 0.93 | 8 | |  |  |
|  | *cmf*KO |  |  | 7.92 ± 1.40 | 6 | |  | *****= 0.01 *vs.* CTR |
|  | | | | | | | | |
| **E** | **genotype** | **treat-ment** | **target** | **x-fold mRNA** | **n** | | **statistics** | **P** |
|  | CTR | basal | *Acta2/*  *Hprt* | 257.278±26.208 | 6 | | 2way ANOVA  F_1.20_ = 7.3  p = 0.0137 |  |
|  |  | 8-Br |  | 161.523±13.518 | 6 | |  | **†** = 0.0144 *vs.* basal |
|  | *cmf*KO | basal |  | 193.072±19.940 | 6 | |  |  |
|  |  | 8-Br |  | 180.675±18.306 | 6 | |  | 0.9712 *vs.* basal |
|  | CTR | basal | *Col1a1/ Hprt* | 152.842±25.984 | 5 | | 2way ANOVA  F_1.18_ = 7.540  p = 0.0133 |  |
|  |  | 8-Br |  | 71.628±11.848 | 5 | |  | **†** = 0.0191 *vs.* basal |
|  | *cmf*KO | basal |  | 86.482±13.311 | 6 | |  |  |
|  |  | 8-Br |  | 76.102±13.766 | 6 | |  | 0.9665 *vs.* basal |
|  | CTR | basal | *Fn1/*  *Hprt* | 13.170±2.892 | 4 | | 2way ANOVA  F_1.15_ = 5.836  p = 0.0289 |  |
|  |  | 8-Br |  | 4.620±0.474 | 5 | |  | **†** = 0.0479 *vs.* basal |
|  | *cmf*KO | basal |  | 8.956±2.173 | 5 | |  |  |
|  |  | 8-Br |  | 8.192±1.776 | 5 | |  | 0.9999 *vs.* basal |
|  | CTR | basal | *Tgfβ1*  */Hprt* | 0.697±0.117 | 6 | | 2way ANOVA  F_1.19_ = 0.6439  p = 0.4322 |  |
|  |  | 8-Br |  | 0.724±0.078 | 5 | |  | 0.9996 *vs.* basal |
|  | *cmf*KO | basal |  | 1.261±0.260 | 5 | |  |  |
|  |  | 8-Br |  | 0.934±0.137 | 5 | |  | 0.5984 *vs.* basal |
|  | CTR | basal | *IL6*  */Hprt* | 1.160±0.2176 | 6 | | 2way ANOVA  F_1.18_ = 4.324  p = 0.0521 |  |
|  |  | 8-Br |  | 1.807±0.324 | 6 | |  | 0.2083 *vs.* basal |
|  | *cmf*KO | basal |  | 1.220±0.144 | 5 | |  |  |
|  |  | 8-Br |  | 1.548±0.146 | 5 | |  | 0.7800 *vs.* basal |

|  | | | | | | |
| --- | --- | --- | --- | --- | --- | --- |
| | **G** | **genotype** | **treatment** | **pVASP/VASP ratio** | **n** | **statistics** | **P** | | --- | --- | --- | --- | --- | --- | --- | | CTR | basal | 0.227±0.053 | 5 | 2way ANOVA  F_1.16_ = 28.20  **p = <0.0001**  (**treatment**)  2way ANOVA  F_1.16_ = 7.63  ***p = <0.013**  (**genotype**) |  | | *cmf*KO | basal | 0.201±0.018 | 5 |  | | CTR | 8-Br | 0.522±0.047 | 5 | **#** = 0.0004 *vs.* CTR basal | | *cmf*KO | 8-Br | 0.329±0.032 | 5 | 0.1490 *vs.* *cmf*KO basal  ***** = 0.0158 *vs.* CTR 8-Br | | | | | | | |
|  | | | | | | |
| **I** | **genotype** | **treatment** | **EF** [%] | **n** | **statistics** | **p** |
|  | CTR | +TAM | 58.56 ± 1.73 | 8 | 2way ANOVA  F_1,28_ = 4.09  p = 0.05  (**treatment**)  2way ANOVA  F_1,28_ = 2.27  p = 0.14  (**genotype**) |  |
|  | *cmf*KO |  | 60.90 ± 2.57 | 7 |  | 0.95 *vs.* CTR+TAM |
|  | CTR | +TAM + Ang II | 59.34 ± 3.57 | 8 |  | 0.99 *vs.* CTR+TAM |
|  | *cmf*KO |  | 48.31 ± 3.15 | 9 |  | **†** = 0.02 *vs.* *cmf*KO+TAM; |
|  |  |  |  |  |  | ***** = 0.04 vs CTR+TAM+Ang II |
|  | | | | | | |
| **J** | **genotype** | **treatment** | **FS** [%] | **n** | **statistics** | **p** |
|  | CTR | +TAM | 30.77 ± 1.20 | 8 | 2way ANOVA  F_1,28_ = 4.523  **p = 0.04**  **(treatment)**  2way ANOVA  F_1,28_ = 2.210  p = 0.14  (genotype) |  |
|  | *cmf*KO |  | 32.36 ± 1.70 | 7 |  | 0.93 *vs.* CTR+TAM |
|  | CTR | +TAM + Ang II | 31.20 ± 2.32 | 8 |  | 0.99 *vs.* CTR+TAM |
|  | *cmf*KO |  | 24.19 ± 1.79 | 9 |  | **†** = 0.02 *vs.* *cmf*KO+TAM; |
|  |  |  |  |  |  | ***** = 0.04 vs CTR+TAM+Ang II |
